# Reengineering statin therapy to protect skeletal muscle: nanocarrier strategies for mitigating mitochondrial dysfunction and myotoxicity

**DOI:** 10.1186/s41232-026-00413-9

**Published:** 2026-04-20

**Authors:** Obaydah Abd Alkader Alabrahim, Nageh K. Allam

**Affiliations:** Energy Materials Laboratory (EML), Department of Physics, School of Sciences and Engineering, The American University in Cairo, New Cairo, 11835 Egypt

**Keywords:** Statins, Myopathy, Myocytotoxicity, Nanoparticles, Coenzyme Q10, Selenium, Co-encapsulation, Characterization, Safety, Clinical translational

## Abstract

Statins remain the cornerstone of atherosclerotic cardiovascular disease prevention; however, their long-term clinical utility is frequently limited by skeletal muscle toxicity, ranging from mild myalgia to severe myopathy. Growing evidence implicates mitochondrial dysfunction, oxidative stress, and impaired muscle energy homeostasis as central drivers of statin-induced muscle injury, with important consequences for patient adherence and treatment durability. Emerging nanocarrier-based drug delivery strategies provide an opportunity to address these limitations by reprogramming statin biodistribution, intracellular exposure, and release kinetics in a biologically informed manner. This review critically examines preclinical and early clinical studies of nanoformulated statins, including solid lipid nanoparticles, polymeric and hyaluronic acid–based carriers, nanocrystals, and porous microsponges, across widely prescribed agents such as simvastatin, atorvastatin, and pitavastatin. Across multiple animal models, nanocarrier-mediated delivery consistently enhances hepatic targeting while attenuating systemic peak exposure, leading to marked reductions in biochemical, histopathological, and functional indicators of skeletal muscle injury relative to conventional formulations. Co-encapsulation strategies incorporating mitochondrial-supportive agents, such as coenzyme Q10 or selenium, further amplify muscle protection while enabling dose reduction. Mechanistically, these protective effects are associated with preservation of mitochondrial respiratory capacity, suppression of reactive oxygen species generation, and attenuation of pro-inflammatory signaling within muscle tissue, pathways directly implicated in muscle degeneration and impaired repair. By integrating molecular mechanisms with translational considerations, this review positions nanocarrier-enabled statin delivery as a promising strategy to decouple lipid-lowering efficacy from muscle toxicity, with broader implications for safeguarding skeletal muscle health and function during chronic pharmacotherapy.

## Introduction

Cardiovascular diseases (CVDs) remain the leading cause of mortality and disability worldwide, imposing a substantial burden on global healthcare systems and economies. According to the World Health Organization (WHO, 2025), CVDs accounted for approximately 19.8 million deaths in 2022, nearly 32% of all global mortality, with more than 75% of these deaths occurring in low- and middle-income countries [1, 2]. Consistently, the World Heart Federation’s *World Heart Report 2023* estimated 20.5 million CVD-related deaths in 2021 and highlighted a concerning plateau in mortality reduction despite advances in prevention and treatment [3].

Among CVDs, atherosclerotic cardiovascular disease (ASCVD) is the most prevalent and clinically significant subtype. It is characterized by progressive lipid deposition, chronic vascular inflammation, and plaque formation, ultimately leading to myocardial infarction, ischemic stroke, and peripheral artery disease. Current management strategies for ASCVD include lifestyle modification, lipid-lowering pharmacotherapy, and revascularization procedures such as percutaneous coronary intervention or coronary artery bypass grafting [2, 4, 5]. However, interventional approaches are invasive and costly, while pharmacological therapies are frequently limited by long-term adherence challenges, drug intolerance, and residual cardiovascular risk.

Statins (3-hydroxy-3-methylglutaryl–coenzyme A reductase inhibitors) remain the cornerstone of lipid-lowering therapy and ASCVD prevention. Their widespread use has led to marked reductions in low-density lipoprotein cholesterol (LDL-C), plaque stabilization, and significant declines in cardiovascular morbidity and mortality [6, 7]. Despite these benefits, statin therapy is often compromised by muscle-related adverse effects, collectively referred to as statin-associated myopathy (SAM). These effects range from mild myalgia to severe myopathy and, in rare cases, rhabdomyolysis, resulting in treatment discontinuation in up to 10–15% of patients [7, 8]. Lipophilic statins, such as simvastatin and atorvastatin, are particularly associated with a higher incidence of muscular symptoms, likely due to their enhanced penetration into extrahepatic tissues [9, 10].

**Fig. 1:**
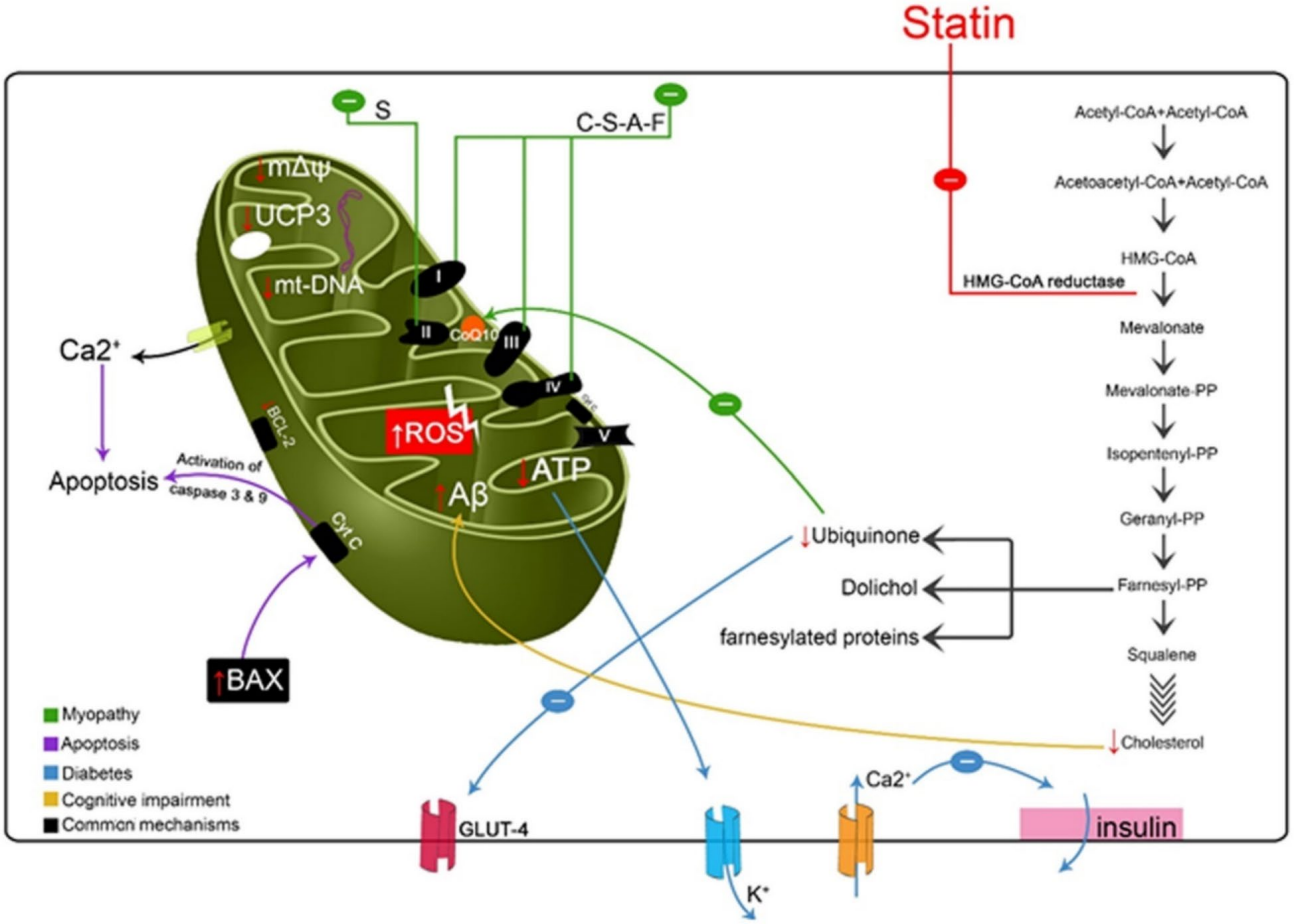
Statins inhibit HMG-CoA reductase, blocking the mevalonate pathway and reducing synthesis of cholesterol, CoQ10, and prenylated proteins. These changes impair mitochondrial potential, decrease CoQ10 and GLUT-4 expression, increase ROS, and trigger apoptosis via BAX and cytochrome c. They also disrupt Ca^2^⁺ homeostasis, downregulate UCP3 and β-oxidation, and raise amyloid-β levels, leading to reduced ATP and muscle injury across multiple statins. *A* atorvastatin, *C* cerivastatin, *F* fluvastatin, *S* simvastatin, *HMG*-*CoA* β-hydroxy β-methylglutaryl-coenzyme A, *CoQ10* coenzyme Q10, *ROS* reactive oxygen species, *UCP3* uncoupling protein 3, *Aβ* amyloid-β, *mtDNA* mitochondrial DNA. Pathway labels: (i) Loss of mitochondrial potential; (ii) CoQ10/GLUT-4 reduction; (iii) ROS increase and apoptosis; (iv) Ca^2^⁺ imbalance; (v) Mitochondrial depletion; (vi) UCP3 loss and β-oxidation decrease; (vii) Aβ accumulation; (viii) Respiratory-chain inhibition. Reproduced from ref. [4]. Copyright 2021 John Wiley & Sons

**Fig. 2:**
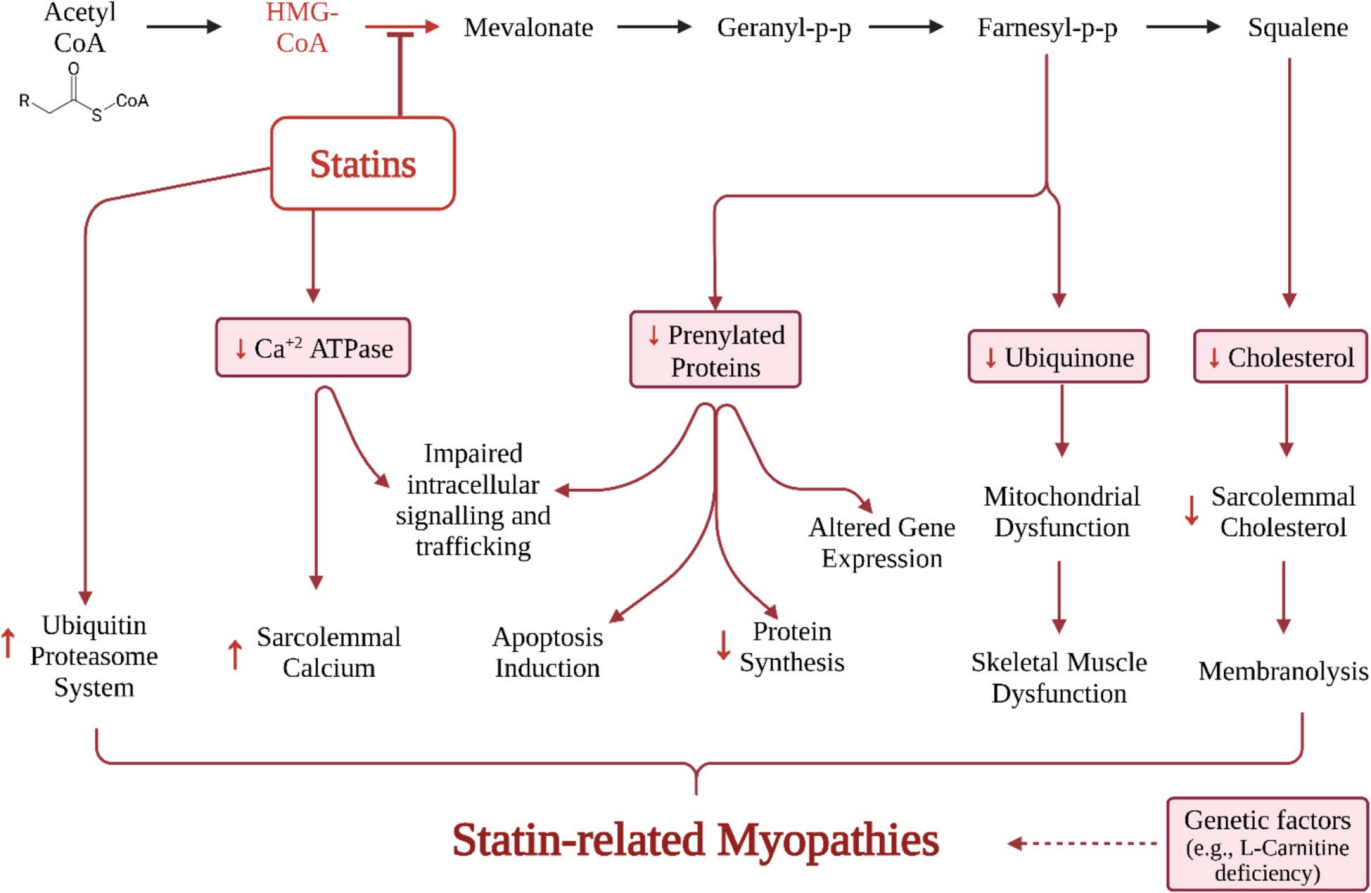
Statins inhibit HMG-CoA reductase, suppressing the mevalonate pathway and reducing synthesis of cholesterol, prenylated proteins, and ubiquinone (CoQ10). This depletion disrupts cellular signaling, protein synthesis, and mitochondrial energy production, leading to elevated ROS, impaired Ca^2^⁺ homeostasis, and activation of the ubiquitin–proteasome system. These combined effects trigger mitochondrial and membrane dysfunction and contribute to statin-induced myopathy, particularly in genetically or metabolically susceptible individuals [2]. Figure drawn using Biorender

The pathogenesis of SAM is multifactorial and not fully elucidated; however, substantial evidence implicates mitochondrial dysfunction, oxidative stress, and calcium dysregulation as central mechanisms (Fig. 1). Inhibition of the mevalonate pathway by statins not only suppresses cholesterol synthesis but also reduces the biosynthesis of essential intermediates, including ubiquinone (coenzyme Q10) and isoprenoids required for protein prenylation. Depletion of CoQ10 impairs mitochondrial electron transport and ATP production, while increased reactive oxygen species (ROS) promotes oxidative damage, inflammation, and apoptotic signaling in skeletal muscle cells (Fig. 2) [11–14]. Additional contributors include disrupted calcium homeostasis, compromised sarcolemmal integrity, and impaired signaling through prenylated proteins such as RhoA and Rac1, collectively hindering muscle repair and regeneration. Genetic polymorphisms (e.g., *SLCO1B1*) and drug–drug interactions that elevate systemic statin exposure further exacerbate susceptibility to myotoxicity [1–5].

From a formulation perspective, free statin molecules are characterized by poor aqueous solubility, extensive first-pass hepatic metabolism, and highly variable oral bioavailability, necessitating relatively high doses to achieve therapeutic plasma concentrations [12, 15–17]. Elevated systemic exposure consequently increases the likelihood of extrahepatic distribution, particularly to skeletal muscle tissue. This effect is especially pronounced for lipophilic statins such as simvastatin, which readily diffuse across muscle cell membranes [18, 19]. Such non-selective tissue distribution is widely recognized as a major contributor to statin-associated myotoxicity, particularly at higher doses or when co-administered with CYP3A4 inhibitors that further increase systemic exposure [10, 11].

Accordingly, the pharmacokinetic (PK) heterogeneity among statins plays a critical role in determining their muscle toxicity profiles. Lipophilic statins, including simvastatin and atorvastatin, exhibit greater passive diffusion into skeletal muscle, whereas hydrophilic agents such as pravastatin and rosuvastatin demonstrate enhanced hepatoselectivity and a comparatively lower risk of myotoxicity. These distinctions underscore the urgent need for rational formulation strategies that preferentially localize statin activity to hepatic tissues while minimizing skeletal muscle exposure[10, 11].

As summarized in Table 1, simvastatin’s high lipophilicity, extensive CYP3A4-mediated metabolism, and low oral bioavailability make it an especially compelling candidate for reformulation into targeted nanoscale delivery systems designed to reduce systemic circulation and muscle accumulation while preserving lipid-lowering efficacy.

**Table 1:** Comparative PK and physicochemical properties of major statins relevant to myotoxicity and nanoformulation suitability. Data summarized and adapted from refs. [6, 7], with supporting information from additional pharmacokinetic references cited in text

Statin	Solubility	Bioavailability (%)	Half-life (h)	Metabolism (major CYP)	Protein binding (%)	Myopathy risk	Remarks
Simvastatin	Lipophilic	< 5	1–2	CYP3A4	95	High	Lactone form Easily enters muscle tissue High myotoxic risk
Atorvastatin	Lipophilic	12–14	14	CYP3A4	≥ 90	Moderate–High	Long half-life Partial hepatic first-pass metabolism
Pitavastatin	Lipophilic	50–60	11–12	CYP2C9	96	Moderate	Limited CYP metabolism Better bioavailability Newer agent
Rosuvastatin	Hydrophilic	20	19	CYP2C9/2C19	89	Low	Hydrophilic, reduced muscle penetration Lower Myotoxicity
Pravastatin	Hydrophilic	17–18	1–2	Sulphation	50	Low	Minimal CYP metabolism Lower myopathy incidence

Recent advances in nanomedicine offer innovative strategies to modulate the pharmacokinetics and tissue distribution of statins. Nanoparticles, liposomes, and lipid- or polymer-based carriers enable controlled drug release, enhanced targeting to hepatocytes or atherosclerotic plaques, and reduced peak systemic concentrations, thereby potentially minimizing skeletal muscle exposure [20–22]. In addition, nanocarrier encapsulation improves statin solubility and stability, allowing lower therapeutic doses while preserving lipid-lowering efficacy [22]. Some platforms further incorporate the co-delivery of mitochondrial protectants, such as antioxidants or coenzyme Q10, directly addressing key biochemical mechanisms underlying statin-induced myotoxicity [23]. Notably, simvastatin nanoformulations, including hyaluronan-based nanoparticles, lipid microsponges, and polymeric nanocarriers, have demonstrated significant reductions in cytoskeletal damage, oxidative stress, and apoptotic signaling in skeletal muscle models [23–26]. These findings support the premise that nanotechnology can overcome the dose-limiting myopathy that constrains conventional statin therapy.

In this context, the present review critically evaluates nanoformulated simvastatin systems developed to mitigate myopathy and myocytotoxicity, comparing their performance with free drug formulations and highlighting mechanistic insights from preclinical investigations. Nano-enabled delivery of other statins, particularly atorvastatin and pitavastatin, is also briefly discussed where safety enhancement has been reported. Emphasis is placed on formulation design, physicochemical properties, biological evaluation, and the capacity of nanocarriers to address molecular pathways implicated in statin-induced muscle toxicity.

## Mechanistic basis of statin-induced myopathy: linking pharmacology to nano-strategies

SAM encompasses a spectrum of muscle disorders ranging from mild myalgia to severe rhabdomyolysis, potentially compromising adherence and diminishing therapeutic benefit of statins [8, 9]. The likelihood of SAM reflects both patient-specific factors and drug physicochemical properties [10, 11]. Among these, lipophilicity and PKs are decisive determinants of extrahepatic exposure and thus muscle toxicity. Lipophilic statins (e.g., simvastatin, atorvastatin) passively diffuse into skeletal muscle to a greater extent than hydrophilic analogs (e.g., pravastatin, rosuvastatin), which rely more on hepatic uptake via organic anion transporting polypeptides (OATP) transporters. This difference forms the pharmacological foundation of myotoxic potential [7, 12–14].

### Pharmacology and tissue distribution

Statins inhibit HMG-CoA reductase in hepatocytes, suppressing cholesterol synthesis and upregulating LDL receptors, the pharmacodynamic basis of LDL-C reduction. However, the same systemic exposure that enables LDL-C lowering also allows statins distribution to reach non-hepatic tissues [10, 27, 28]. Lipophilic statins readily permeate muscle membranes, accumulating intracellularly and predisposing cells to toxicity, whereas hydrophilic statins display greater hepatoselectivity due to active uptake transport [12–14].

Beyond passive diffusion driven by lipophilicity, statin disposition in skeletal muscle is influenced by transporter-mediated uptake mechanisms. Several OATPs and organic cation transporters have been detected in skeletal muscle and may facilitate intracellular statin accumulation under certain physiological and pharmacological conditions. Genetic polymorphisms affecting hepatic transporter activity, most notably *SLCO1B1* variants, markedly increase systemic statin exposure and predispose patients to myopathy [15, 16, 29, 30].

Pharmacological inhibition of muscle-expressed statin transporters has been proposed as a theoretical strategy to reduce myotoxicity risk; however, such an approach lacks tissue specificity and may disrupt hepatic uptake or systemic clearance, potentially exacerbating plasma exposure. In contrast, nanocarrier-based delivery provides a spatial and kinetic strategy to modulate biodistribution while preserving endogenous transporter function.

PK factors such as low oral bioavailability, high protein binding, short half-life, and extensive CYP3A4 metabolism (as in simvastatin) can lead to temporarily high peak plasma levels (Cmax) or metabolites’ rises that increase muscle exposure [7, 17]. Hence, formulation strategies that attenuate systemic peaks, prolong release, or enhance hepatic targeting are mechanistically justified to lower myopathy risk [7, 12–14, 17].

### Molecular mechanisms of muscle injury

**Fig. 3:**
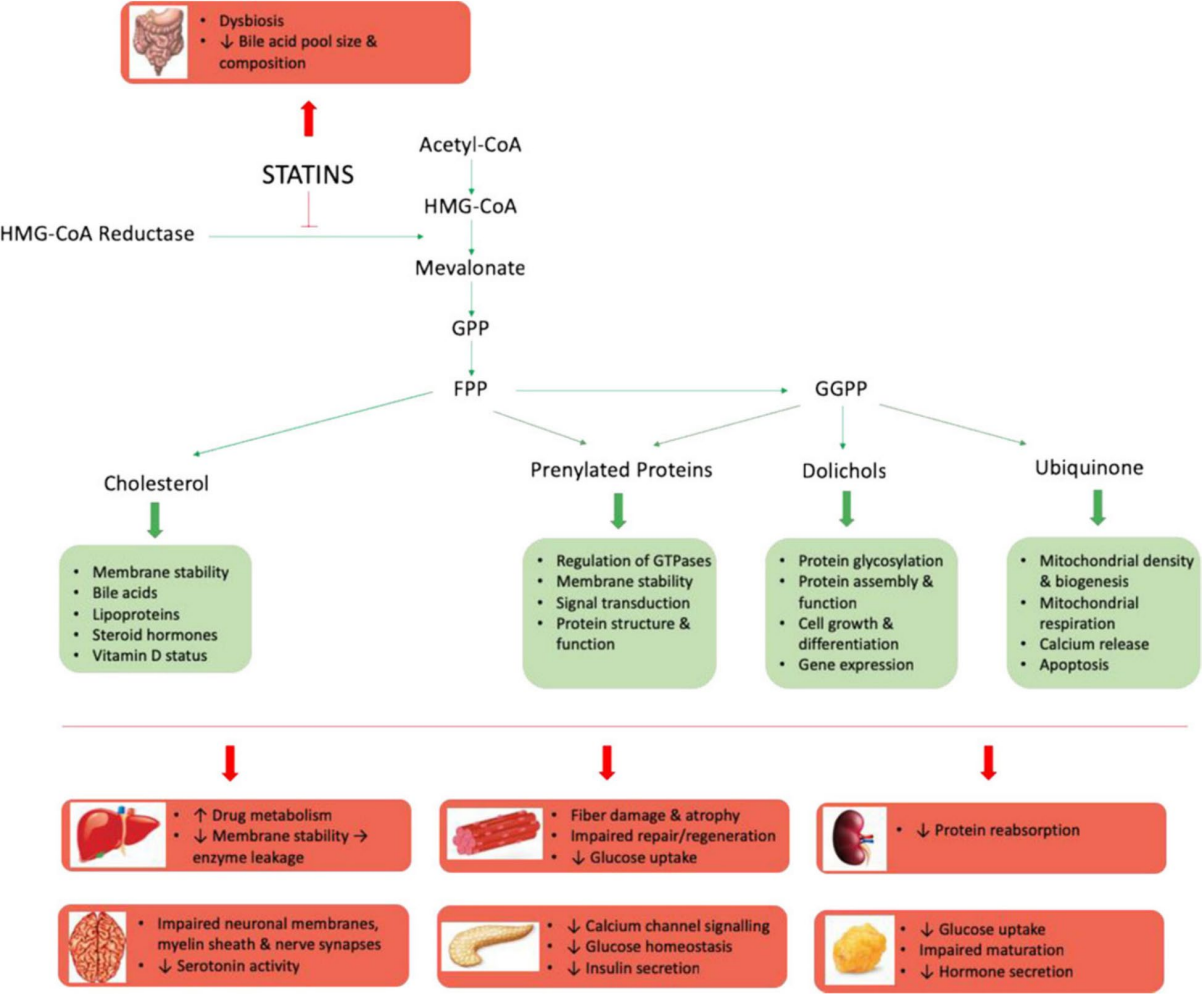
Schematic representation of the mevalonate pathway and potential mechanisms contributing to statin toxicity. Inhibition of HMG-CoA reductase by statins suppresses downstream synthesis of cholesterol, prenylated proteins, dolichols, and ubiquinone (CoQ10), resulting in mitochondrial dysfunction, impaired membrane stability, and myotoxicity. *FPP* farnesyl pyrophosphate, *GGPP* geranylgeranyl pyrophosphate, *GPP* geranyl pyrophosphate, *HMG-CoA* hydroxymethylglutaryl-coenzyme A. Reproduced from ref. [18]. Copyright 2019 American Heart Association

SAM pathogenesis is multifactorial, and the principal mechanisms, supported by experimental, in vitro, biopsy, and pharmacogenetic data, can be summarized as follows (Fig. 3):

- Mitochondrial dysfunction and CoQ10 depletion. Statins reduce mevalonate-derived isoprenoid and ubiquinone (CoQ10) synthesis, impairing electron transport and ATP production. Depleted CoQ10 disrupts oxidative phosphorylation, elevates reactive oxygen species (ROS), and predisposes muscle fibers to necrosis. Muscle biopsies and in vitro myotube studies document mitochondrial structural and functional impairments after statin exposure [19–24].
- Oxidative stress, apoptosis, and impaired muscle repair. Elevated ROS within myocytes leads to oxidative damage of proteins and membranes and activation of apoptotic pathways (caspases). Concomitant mitochondrial dysfunction and increased intracellular Ca^2^⁺ amplify proteolytic enzyme activation (calpains) and cytoskeletal breakdown, further impairing muscle integrity and regeneration [10, 20, 25].
- Disrupted protein prenylation and cellular signaling. By inhibiting the mevalonate pathway, statins reduce farnesyl and geranylgeranyl pyrophosphates necessary for prenylation of small GTPases (Ras/Rho/Rac). Loss of prenylation alters intracellular signaling, cytoskeletal organization, and membrane maintenance that are vital for muscle cell survival and mechanical resilience [20, 26, 31].
- Sarcoplasmic reticulum (SR) calcium leak and ryanodine receptor (RyR1) dysfunction. Statin exposure perturbs calcium homeostasis through RyR1 destabilization and associated regulatory proteins, promoting SR Ca^2^⁺ leak and sustained cytosolic Ca^2^⁺ elevation, which trigger degradation and apoptosis, a mechanism that may help explain acute myalgia and susceptibility to more severe myotoxicity in some patients [10, 32].
- Genetic and transporter influences. Pharmacogenomics links genetic variations to statin adverse effects, most commonly myopathy. Key genes involved include *SLCO1B1* (c.521T > C polymorphism), which encodes a protein that transports statins into liver cells and, therefore, certain variants of this gene can decrease the protein’s function, resulting in greater levels of statins in the blood and increasing their associated-myopathic adverse effects. Other polymorphisms in CYP2C9 and CYP3A4 further modulate statin metabolism, resulting in higher systemic exposure and adverse effects of statins [13, 14, 32, 33]. Genetic testing can help predict individual risk and guide statin selection or dosing to improve safety. Therefore, carriers of risk alleles have higher plasma statin concentrations and a markedly increased risk of SAM [14, 29]. Thus, pharmacogenomics highlights that systemic exposure, not inherent drug toxicity, is the primary risk driver, supporting delivery systems that minimize peripheral distribution and muscles exposure.

**Fig. 4:**
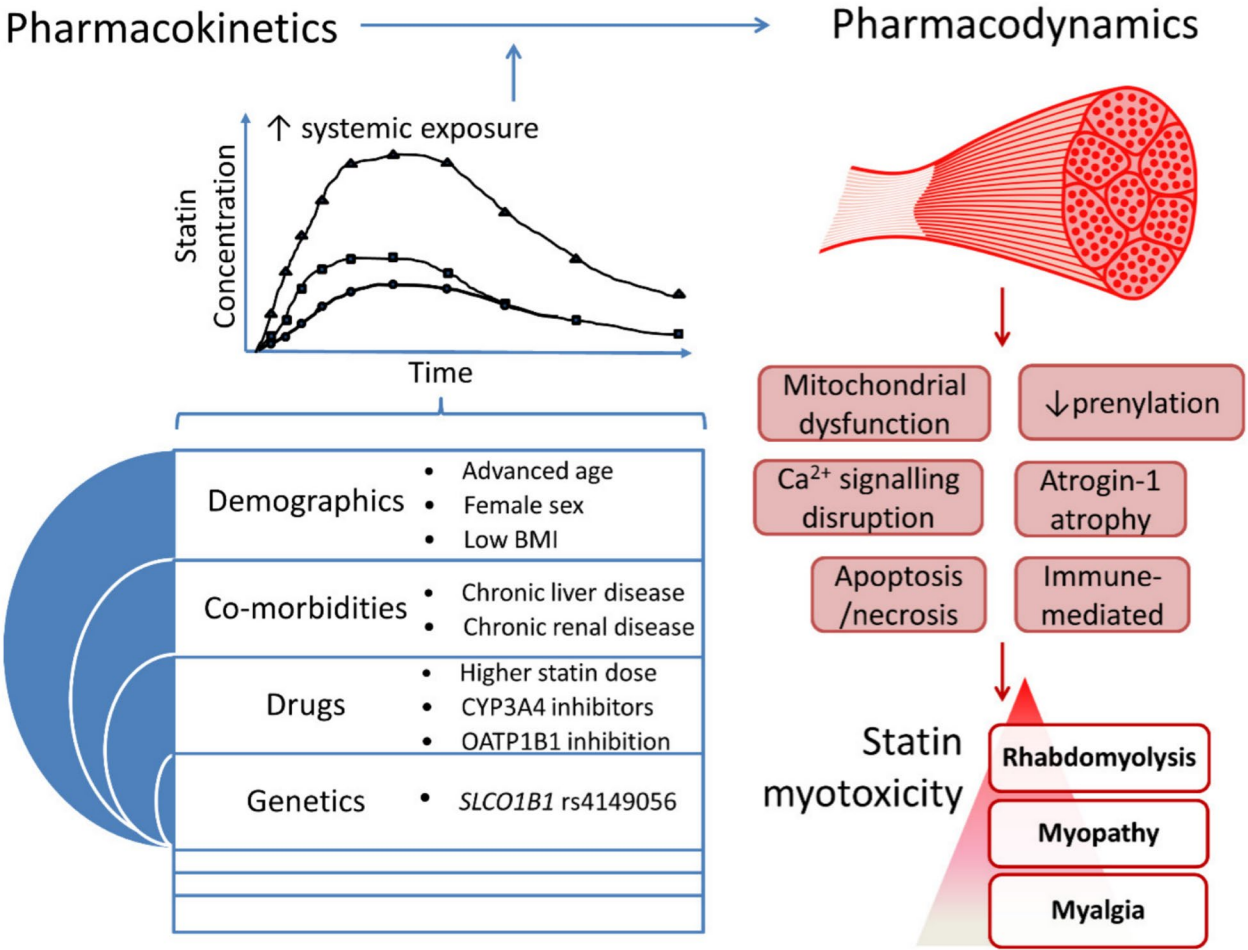
Overview of PK, pharmacodynamic, and patient-related contributors to statin-induced myotoxicity. Factors such as age, genetic variants (e.g., *SLCO1B1* polymorphisms), comorbidities, and drug interactions increase systemic statin exposure and predispose to mitochondrial dysfunction, impaired prenylation, and muscle injury leading to myalgia, myopathy, and rhabdomyolysis. Reprinted from ref. [34]. Copyright 2019 MDPI

Collectively, these factors determine the extent of extrahepatic statin exposure and thus the likelihood of mitochondrial injury and muscle toxicity (Fig. 4).

Statin-associated myopathy extends beyond acute myofiber injury and has important implications for skeletal muscle regeneration. Efficient muscle repair relies on intact mitochondrial bioenergetics, tightly regulated inflammatory signaling, and activation of satellite cells that coordinate myofiber regeneration. Experimental studies demonstrate that statin-induced mitochondrial dysfunction, excessive ROS production, and apoptotic activation impair myoblast proliferation and differentiation, ultimately delaying muscle regeneration and functional recovery [19, 35–37]. Moreover, chronic inflammatory activation within muscle tissue further disrupts regenerative signaling pathways essential for myofiber repair [37–39].

Emerging evidence suggests that nanocarrier-based statin delivery may indirectly preserve regenerative capacity by limiting mitochondrial injury, attenuating oxidative stress, and reducing pro-inflammatory signaling. Preservation of myofiber architecture reduced apoptotic indices, and improved contractile performance observed in nano-statin systems strongly imply a microenvironment more conducive to effective muscle repair. Although current studies rarely quantify classical regeneration markers such as Pax7, MyoD, or myogenin, the structural and functional protection afforded by nanocarriers highlights a promising regenerative advantage that warrants direct investigation in future work.

### From mechanisms to formulation: how nanosystems interrupt the toxic cascade

Given the mechanisms above, three formulation-level strategies emerge as directly relevant to mitigate SAM:

- Lowering peak systemic exposure (reduced Cmax/controlled release). Controlled-release lipid, polymeric, and other nanocarriers smooth absorption, reducing transient plasma spikes that cause mitochondrial overload and ROS generation [39–44].
- Preferential hepatic or vascular targeting. Ligand modification (e.g., hyaluronan coatings, apolipoprotein mimicry) or size/charge tuning enhances hepatic localization while minimizing muscle exposure [14, 33, 42–47].
- Co-delivery of protective agents. Co-encapsulation of antioxidants or mitochondrial stabilizers (e.g., CoQ10, selenium, and other ROS scavengers) reduces statin toxicity, while protecting mitochondria against ROS and preserving ATP generation even if minor drug exposure occurs [14, 33, 40, 47].

These complementary strategies translate the mechanistic understanding of SAM into rational nanoformulation design. Recent studies demonstrate that such systems reduce serum creatine kinase (CK) levels, preserve myofiber morphology, and attenuate apoptosis compared to free statins [14, 33, 39–42].

## Nanotechnology-based strategies to mitigate statin-induced myopathy

Nanotechnology provides multiple and complementary platforms to reduce statin myotoxicity, while modulating kinetics to limit peak muscle exposure, maximize targeting to the liver, and co-delivering mitochondrial protectants [17, 48–51]. Several preclinical studies have explored whether nano- and micro-formulations of simvastatin can reduce statin-associated myotoxicity by altering PKs, biodistribution, or the local biochemical milieu.

**Fig. 5:**
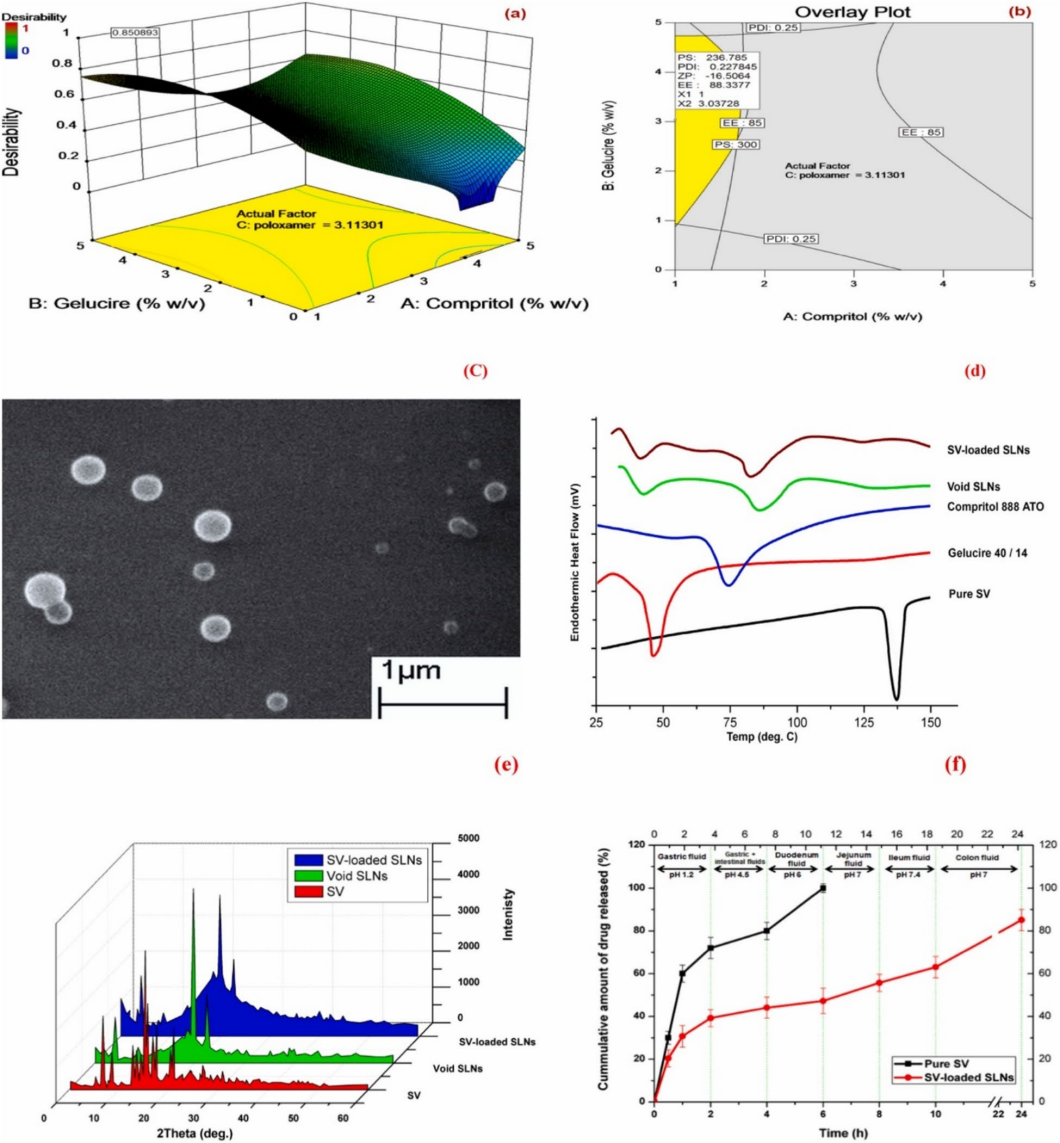
Optimization and characterization of simvastatin-loaded SLNs. **a** 3D surface plot showing the numerical optimization outcome; **b** contour plot displaying the graphical optimization response; **c** SEM image illustrating surface morphology of the optimized SV SLNs; **d** DSC thermograms comparing pure SV, Gelucire 40/14, Compritol 888 ATO, blank SLNs, and optimized SV SLNs; **e** XRD patterns of pure SV, blank SLNs, and optimized SV SLNs; **f** cumulative release profiles of pure SV and optimized SV SLNs under different gastrointestinal pH conditions. Reproduced from ref. [41]. Copyright 2021 Elsevier

Lipid carriers improve solubility of lipophilic drugs, permit controlled drug release, and can be tuned to reduce burst release. They also resemble biological lipoproteins and can be functionalized for liver targeting [52–60]. Abo-zalam et al. (2021) developed simvastatin-loaded solid lipid nanoparticles (SV-SLNs) using a hot-melt ultrasonication technique, aiming to enhance bioavailability and reduce the myotoxic and hepatotoxic adverse effects commonly associated with free simvastatin therapy [41]. The formulation process was optimized via a Box–Behnken experimental design to determine the ideal lipid composition and surfactant levels for maximum stability and drug entrapment (Fig. 5). The optimized SV-SLNs exhibited a mean particle size of 255 ± 7.7 nm, a polydispersity index (PDI) of 0.31 ± 0.09, a zeta potential of about − 19.30 ± 3.25 mV, and an entrapment efficiency (EE) of 89.81 ± 2.1%, collectively indicating a uniform and stable nanodispersion suitable for oral delivery and sustained release. Furthermore, XRD, thermal stability (DSC), release profile, and SEM were conducted on the obtained nanoparticles, showing sustained release and promising stability profile (Fig. 5) [41]. The therapeutic and safety performance of SV-SLNs was evaluated in vivo in a metabolic disease context using Wistar albino rats subjected to a high-fat diet (HFD)-induced dyslipidemic model. Sixty rats were randomized into six groups: control, HFD, vehicle (blank SLN), HFD + free simvastatin (20 mg kg⁻^1^), HFD + SV-SLNs (20 mg kg⁻^1^), and HFD + SV-SLNs (5 mg kg⁻^1^). Drug treatment was administered orally for the final 4 weeks of a 16-week feeding regimen. The study investigated a wide spectrum of endpoints including serum lipid profile, liver enzyme activity, CK levels as a marker of myopathy, oxidative stress parameters, muscle histopathology, and caspase-3 expression as an indicator of apoptosis. As expected, HFD-fed rats displayed profound metabolic derangements, hepatocellular perturbation, and evidence of muscle injury. Free simvastatin improved lipid indices but concurrently provoked hepatotoxicity and myofiber degeneration, consistent with known adverse effects of high-dose statin therapy. Conversely, SV-SLNs markedly improved lipid and biochemical parameters while attenuating hepatocellular and skeletal muscle injury [41]. Notably, the lower-dose SV-SLNs group (5 mg kg⁻^1^) restored several biomarkers, including CK, transaminase activity, and oxidative stress indices, to near-control levels, demonstrating a significant dose-sparing effect attributable to enhanced delivery efficiency and sustained release behavior of the lipid nanocarrier [41].

**Fig. 6:**
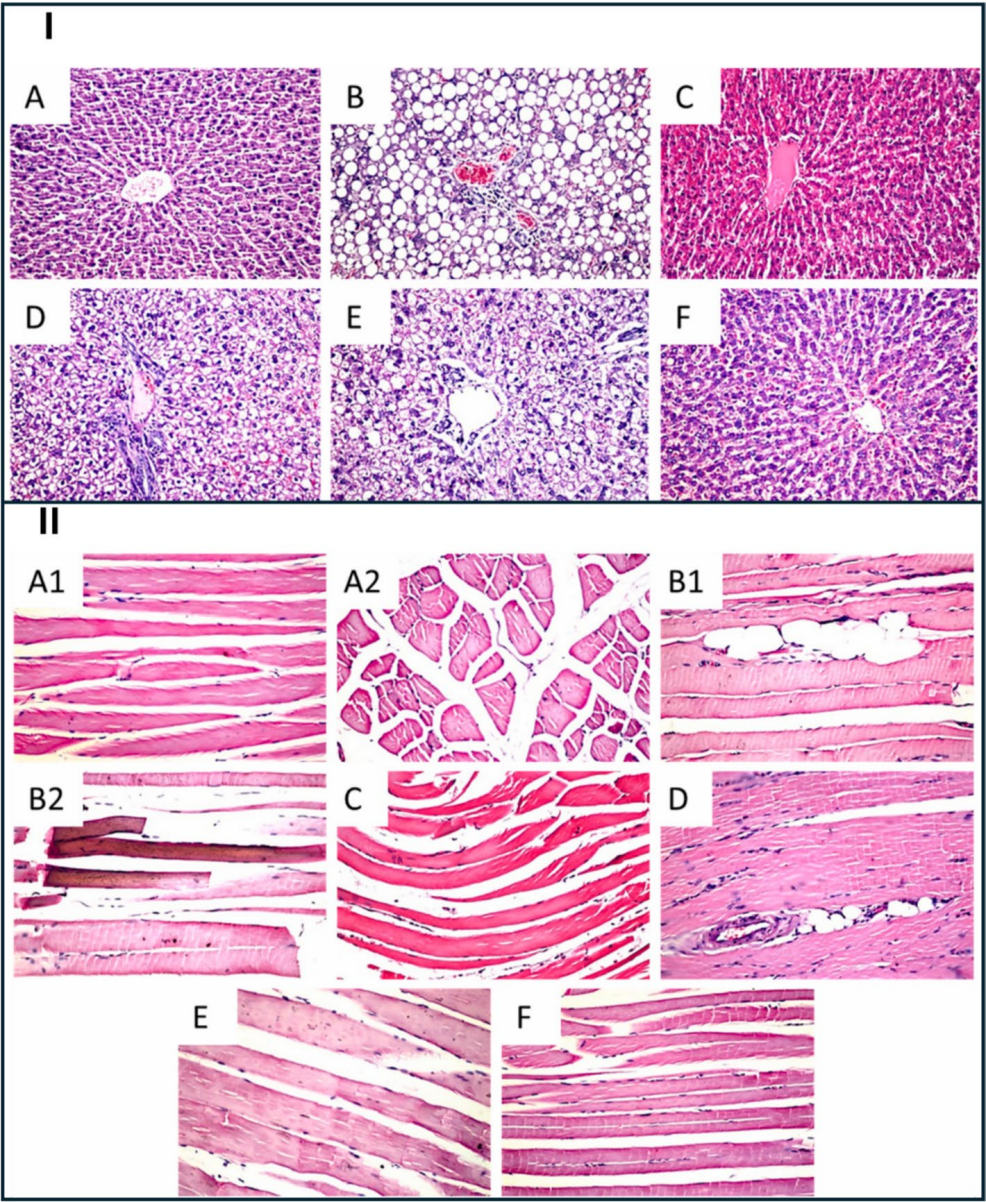
Histological and immunohistochemical evaluation of rat liver and quadriceps tissues after simvastatin treatments. (I) Representative H&E-stained liver sections (× 40): **A** normal control with intact central vein and hepatocytes; **B** hyperlipidemic control showing portal-vein congestion, inflammatory-cell infiltration, and fatty change throughout the parenchyma; C nano-vehicle showing mild congestion in central vein and sinusoids; **D** free simvastatin group exhibiting diffuse vacuolar degeneration with scattered inflammatory infiltration; **E** nano-simvastatin (20 mg/kg) showing vacuolar degeneration in most hepatocytes with limited fatty change; **F** nano-simvastatin (5 mg/kg) showing minimum fatty change with sinusoidal congestion. (II) Representative H&E-stained quadriceps sections (× 40): (A1–A2) normal control displaying intact longitudinal and cross-section muscle bundles; (B1–B2) hyperlipidemic control showing lipid droplets between bundles and Zenker’s necrosis in some fibers; (C) nano-vehicle with preserved normal structure; (D) free simvastatin group showing focal fat cells among fibers; (E) nano-simvastatin (20 mg/kg) and (F) nano-simvastatin (5 mg/kg) demonstrating normal muscle architecture. Reproduced from ref. [41]. Copyright 2021 Elsevier

**Fig. 7:**
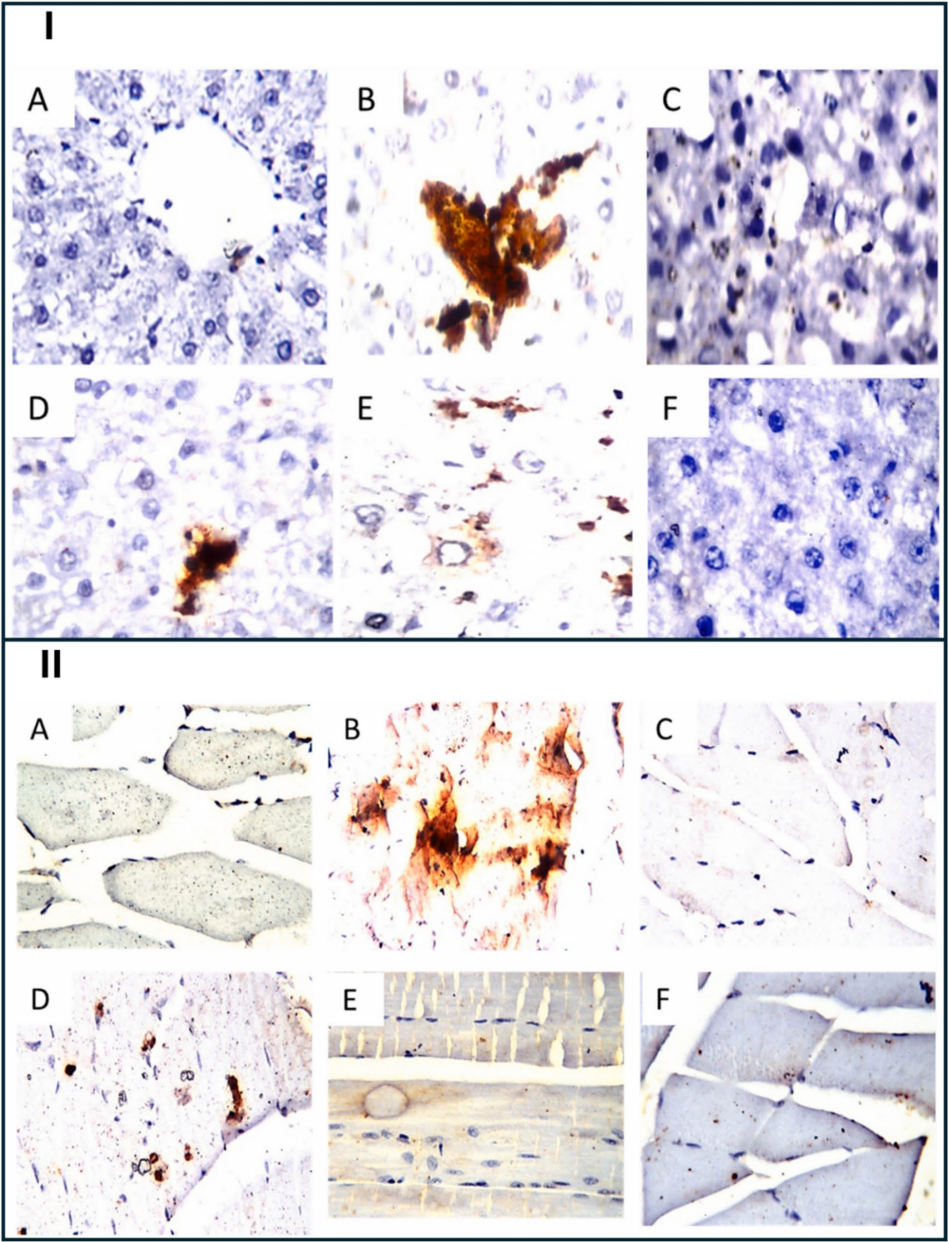
Histological and immunohistochemical evaluation of rat liver and quadriceps tissues after simvastatin treatments. (I) Caspase-3 immunohistochemistry in liver (× 160): marked immunoreactivity (higher caspase activity) in hyperlipidemic (**B**) and free simvastatin (**D**) groups, while normal (**A**), nano-vehicle (**C**), and nano-simvastatin (20 mg/kg, E; 5 mg/kg, F) showed reduced staining comparable to mild activity in free simvastatin group (**D**). Caspase signal appears as dark-brown staining. (II) Caspase-3 immunohistochemistry in quadriceps (× 80): intense staining in hyperlipidemic (**B**) and free simvastatin (**D**) groups compared to lower reactivity in normal (**A**), nano-vehicle (**C**), and nano-simvastatin (20 mg/kg, E; 5 mg/kg, F) groups. Caspase positivity appears as dark-brown staining. Reproduced from ref. [41]. Copyright 2021 Elsevier

Histological assessments further revealed that muscle tissue from the SV-SLN–treated rats maintained normal architecture with minimal infiltration or necrosis, accompanied by reduced expression of caspase-3 compared to the free simvastatin group (Figs. 6 and 7), supporting the hypothesis of decreased apoptosis and improved mitochondrial preservation. Collectively, these outcomes indicate that SV-SLN administration provided a more favorable efficacy–toxicity balance, implying enhanced therapeutic performance even at reduced dosing levels. However, the authors noted that PK data such as plasma Cmax, AUC, or direct tissue concentration measurements were not obtained, leaving the precise mechanistic basis, whether improved distribution, absorption, or depot formation, largely inferred. Additionally, the 4-week therapeutic window within a 16-week disease model restricts conclusions regarding long-term safety, chronic lipid-lowering performance, and reproducibility across species [41]. Despite these limitations, the study provides robust in vivo evidence that lipid-based nanoparticles can enable lower effective simvastatin doses while mitigating myotoxicity and hepatotoxicity markers, reinforcing the translational promise of solid lipid nanoparticles (SLN)-based statin delivery systems and underscoring the need for future PK–pharmacodynamic correlation studies to fully elucidate the mechanism behind reduced tissue toxicity.

**Fig. 8:**
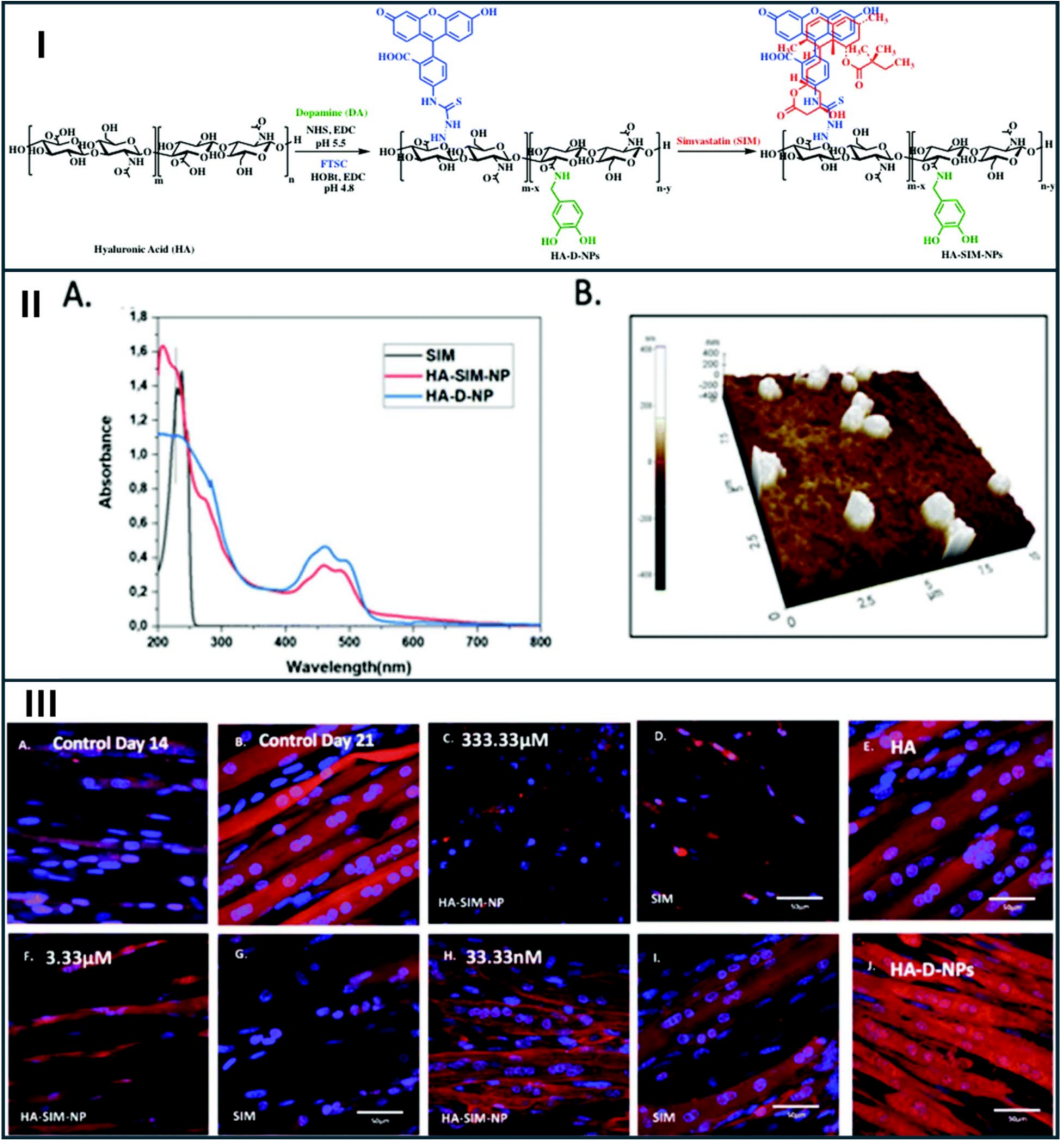
Synthesis, characterization, and biological evaluation of hyaluronic acid–based simvastatin nanoparticles. (I) Schematic illustration of the synthesis and self-assembly of SIM-loaded HA nanoparticles. (II) (**a**) UV–Vis spectra of simvastatin (SIM), unloaded nanoparticles (HA-D-NPs), and nanoparticles loaded with simvastatin (HA-SIM-NPs) recorded in water at 25 °C; (**b**) AFM image showing spherical HA-SIM-NPs (200–300 nm). (III) Fluorescence images of actin (red) and nuclear DNA (blue) staining in tissue-engineered skeletal muscle six days after exposure to aqueous SIM or HA-SIM-NPs. (**A**–**B**) untreated controls at days 14 and 21; (**C**, **F**, **H**) HA-SIM-NP groups, and (**D**, **G**, **I**) free SIM groups treated at 333.33 μM (**C** and **D**), 3.33 μM (**F** and **G**), and 33.33 nM (**H** and **I**); (**E**, **J**) HA and HA-D-NP controls at the highest dose. Images demonstrate dose-dependent myotube preservation, with greater protection at lower concentrations (33.33 nM). Scale bar = 50 μm. Reproduced from ref. [39]. Copyright 2020 RSC

Biodegradable polymers (such as PLGA, PEGylated polymers) allow fine control of release kinetics and surface chemistry [61–64]. Hyaluronan (HA) coatings can promote hepatocyte or macrophage interactions (depending on design) and reduce nonspecific muscle uptake [65, 66]. Jones et al. (2020) developed a hyaluronan-derived nanoparticle system (HA-SIM-NP) for the encapsulation and sustained delivery of simvastatin, aiming to reduce statin-induced myotoxicity and improve myocyte compatibility within a tissue-engineered skeletal muscle (TE SkM) platform [39]. The nanoparticles were obtained by conjugating simvastatin to a HA backbone to yield spherical HA-SIM-NP structures, as confirmed by atomic force microscopy (Fig. 8). The mean hydrodynamic diameter of HA-SIM-NP was approximately 280nm with a zeta potential of − 25.6 mV, indicating stable colloidal behavior, while the unloaded HA carrier exhibited a larger size of ∼ 661 nm prior to drug incorporation [39].

To assess biological outcomes, the HA-SIM-NP formulation was evaluated within a 3-D collagen-based TE SkM model, which serves as a biomimetic in vitro platform for statin-induced myotoxicity screening. Dose–response experiments covered simvastatin concentrations of 33.33 nM, 3.33 µM, and 333.33 µM (the latter causing overt myotube loss and structural ablation) (Fig. 8). Endpoints included morphological assessment of myotube integrity and number, myotube diameter, metabolic viability, contractile and relaxation performance, gene expression analyses (MMP2, MMP9, and myogenin), and histological evaluation. Free simvastatin triggered dose-dependent cytoskeletal disruption, mitochondrial impairment, and decreased tissue contractile responses, whereas HA-SIM-NP markedly ameliorated these deteriorative effects, particularly at intermediate and lower concentrations. Nevertheless, exposure to the highest concentration (333 µM) induced significant cellular and structural damage irrespective of the delivery vehicle [39].

**Fig. 9:**
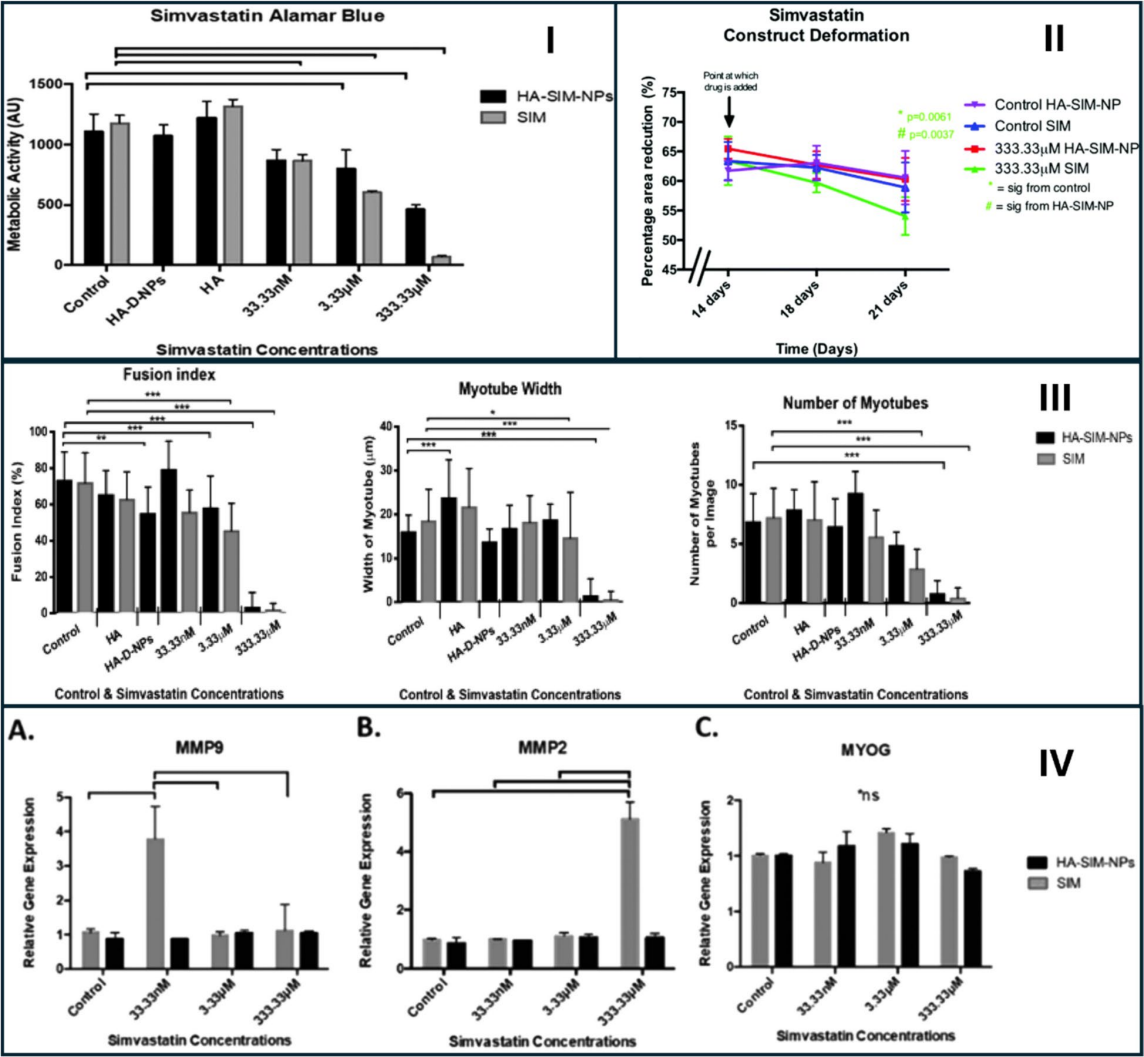
(I) Cell viability of tissue‑engineered skeletal‑muscle constructs six days following aqueous (SIM) or nanoparticle (HA‑SIM‑NP) simvastatin treatment. (II) Muscle‑construct deformation measured immediately (day 14), 3 days (day 18), and 6 days (day 21) following aqueous (SIM) or nanoparticle (HA‑SIM‑NP) simvastatin administration (333.33 μM). (III) Morphological analysis of constructs following simvastatin delivery in nanoparticle (HA‑SIM‑NP) and aqueous (SIM) form at 333 μM, 3.33 μM, and 33.3 nM, along with drug‑free HA‑D‑NPs and HA controls. (IV) mRNA expression of matrix metalloproteinase MMP‑2, MMP‑9, and myogenin 6 days (day 21) following aqueous (SIM) or nanoparticle (HA‑SIM‑NP) treatment. Reproduced from ref. [39]. Copyright 2020 RSC

Consistent with these findings, the HA-SIM-NP-treated tissues maintained more uniform expression patterns of gelatinase enzymes MMP2 and MMP9 (matrix metalloproteinase), and myogenin, indicative of preserved myogenic and regenerative signaling cascades, in contrast to the disrupted expression profiles observed in tissues exposed to free simvastatin (Fig. 9). Overall, these results confirm that the HA-SIM-NP formulation attenuates simvastatin-induced myotoxicity and preserves tissue function in a physiologically relevant 3-D muscle model [39]. However, the study was confined to an in-vitro setting without PK or systemic toxicity data, as noted by the authors, and the highest concentrations examined considerably exceeded therapeutic plasma levels observed clinically. Moreover, given that hyaluronic acid itself possesses inherent pro-regenerative bioactivity within skeletal muscle, part of the observed protective effects might be attributed to HA-mediated signaling rather than solely to altered PK release kinetics. Nonetheless, this work provides valuable design insight by demonstrating that a nanoformulation capable of both controlled simvastatin release and scaffold-associated regenerative support can effectively mitigate statin myotoxicity at the tissue level, encouraging further in vivo evaluation to determine whether such nanocarriers can modulate systemic exposure, reduce myotoxicity risk, and sustain lipid-lowering efficacy.

Porous carriers (microsponges) entrap drug in a sponge-like matrix, enabling prolonged release and reduced peak plasma levels after administration (oral or topical) [67–70]. For simvastatin, microsponges have been formulated to reduce gastrointestinal/systemic peaks associated with myotoxicity. Ali et al. (2023) formulated porous microsponge carriers (FSM series) based on Eudragit RS-100 for the encapsulation and controlled oral delivery of simvastatin, aiming to mitigate the drug’s muscle toxicity through sustained release kinetics and improved local tolerability [40]. The microsponges were prepared using an emulsion–solvent evaporation technique, followed by optimization of key formulation parameters to achieve high entrapment efficiency and favorable release characteristics. Among the prepared formulations, FSM-1 was identified as the optimized system, demonstrating an entrapment efficiency of approximately 82.54 ± 1.27% and a particle size range between 53.80 ± 6.35 and 86.03 ± 4.79 µm. Furthermore, BET surface area and porosity analyses established that FSM-1 exhibited a well-defined porous microarchitecture (with a specific surface area of 16.6 m^2^/g) capable of enabling a zero-order release profile of simvastatin under physiological conditions, thereby supporting a sustained drug release mechanism [40].

**Fig. 10:**
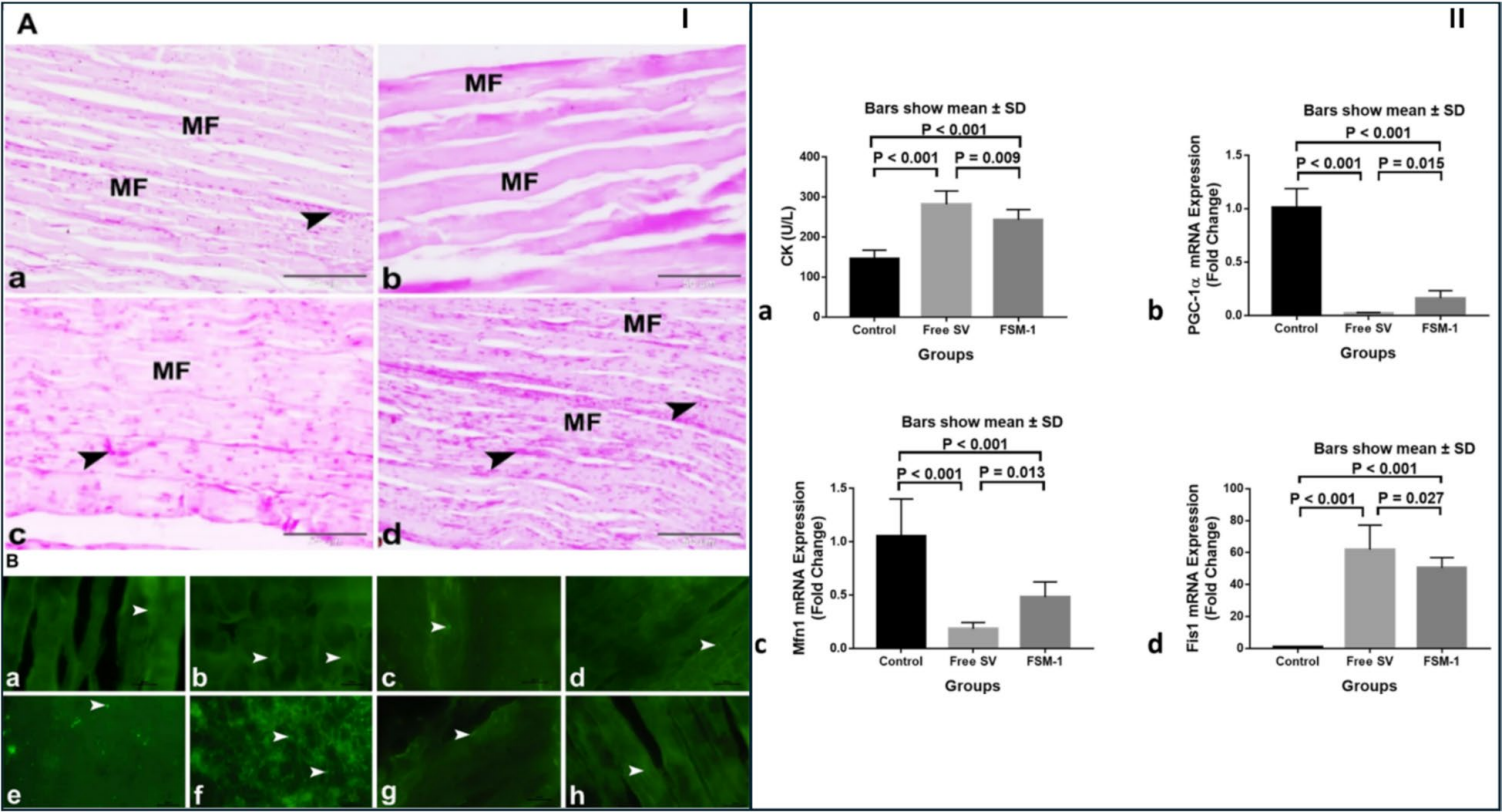
Histological, histochemical, and biochemical evaluation of the protective effect of simvastatin microsponges against myotoxicity in rats. (I) (**A**) PAS staining: (**a**) control: normal structure with abundant PAS glycogen granules in sarcoplasm (arrowhead); (**b**) free SV: marked glycogen depletion; (**c**) FSV‑6: partial restoration; (**d**) FSV‑1: greater restoration of PAS glycogen comparable to control. Scale bar = 50 µm. (**B**) Immunostaining for glutathione reductase (GR, a–d) and superoxide dismutase‑2 (SOD2, e–h): (a,e) control, (b,f) free SV, (c,g) FSV‑6, and (d,h) FSV‑1 group. GR immuno‑expression (arrowheads) was consistent across groups, whereas SOD2 immuno‑expression (arrowheads) was remarkably elevated in the free SV group and significantly reduced in FSV‑6 and FSV‑1 relative to control. Scale bar = 20 µm. **(II)** Serum CK levels (a) and gene expression of PGC‑1α (b), Mfn1 (c), and Fis1 (d) for each group. Reproduced from ref. [40]. Copyright 2023 Nature

**Fig. 11:**
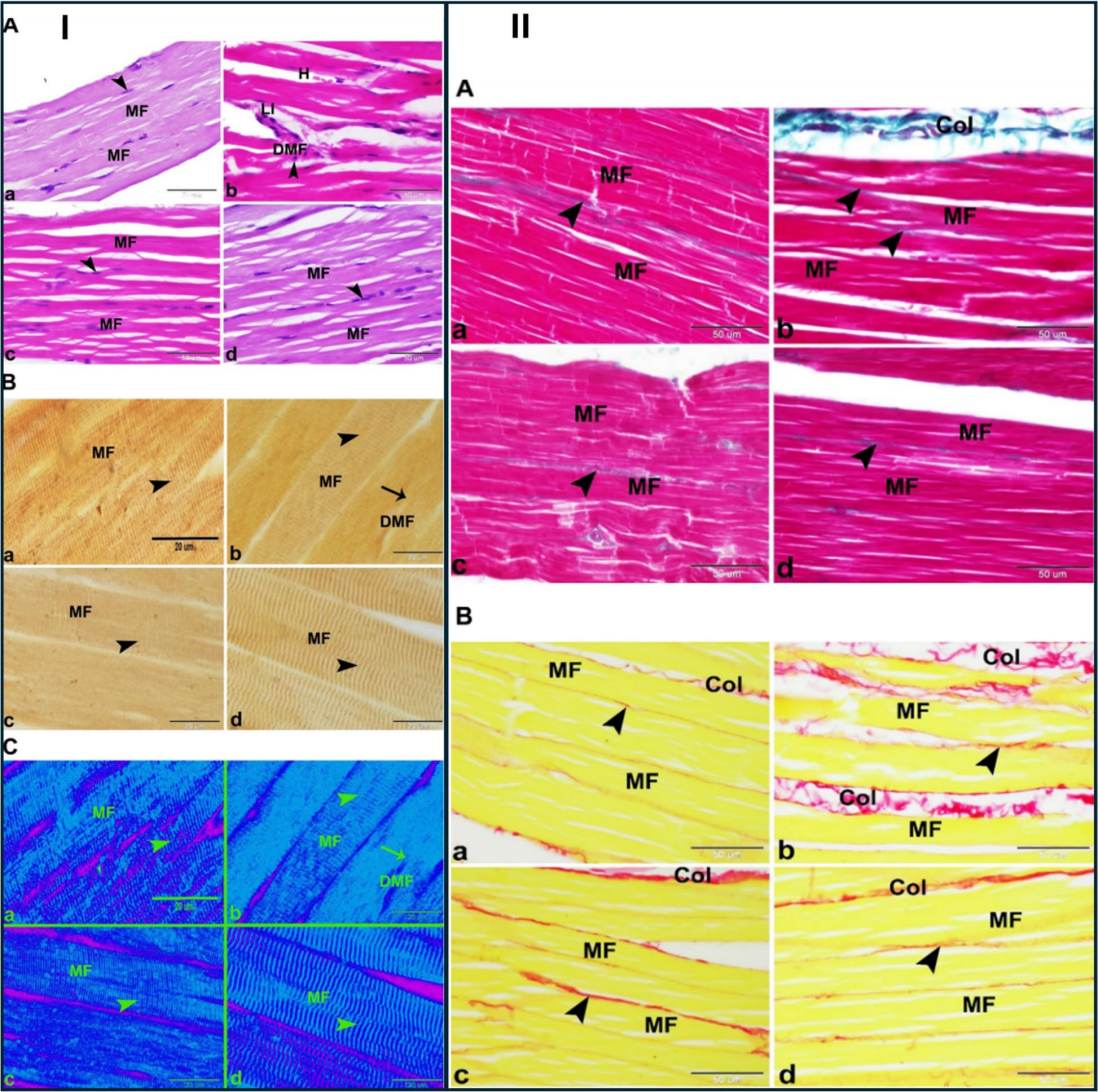
Histological and histochemical evaluation of the protective effect of simvastatin microsponges against myotoxicity in rats. (I) (**A**) H&E-stained skeletal-muscle sections: (a) control: normal parallel muscle fibers (MF) with peripheral nuclei (arrowhead); (b) free SV: degeneration of muscle fibers (DMF) with hyper-eosinophilic sarcoplasm, pyknotic nuclei (arrowhead), hemorrhage (H), and leukocytic infiltration (LI); (c) FSV-6: slight improvement with reduced degeneration; (d) FSV-1: preserved muscle architecture comparable to normal. Scale bar = 50 µm. (**B**) Silver-impregnated sections: (a) control: clear transverse striations (arrowhead); (b) free SV: DMF with loss or ill transverse striations (arrowhead); (c) FSV-6: partial recovery; (d) FSV-1: distinct striations indicating structural restoration. (**C**) Negative images corresponding to panel (B). (II) (A) Masson’s trichrome‑stained sections: (a) control: normal muscle structure appearance accompanied with few collagen fibers in endomysium and perimysium; (b) free SV: increased collagen deposition around fibers (arrowhead; Col), indicating myotoxicity; (c) FSV‑6: moderate reduction; (d) FSV‑1: minimal collagen and myotoxicity comparable to control. (B) Sirius‑red‑stained sections showing similar trends for mature collagen fibers. Scale bar = 50 µm. Reproduced from ref. [40]. Copyright 2023 Nature

The biological performance of the optimized formulation was investigated in vivo using male Wistar rats, divided into control, free simvastatin (SV), and microsponge treatment study groups. Rats received oral doses (20 mg/kg/day) of either the free drug or FSM-1 microsponges, after which serum creatine kinase (CK) levels, skeletal muscle histopathology, and the expression profiles of mitochondrial dynamics–related genes, peroxisome proliferator-activated receptor gamma co-activator 1α (PGC-1α), mitochondrial fusion protein (Mfn1), and mitochondrial fission protein (Fis1), were evaluated as myotoxicity markers (Figs. 10 and 11). Compared to free simvastatin treatment, which caused significant elevation of serum CK (indicative of sarcolemmal damage), morphological degeneration, and inflammatory infiltration in skeletal muscle, administration of FSM-1 microsponges resulted in a markedly attenuated myotoxic response. Specifically, FSM-1 significantly reduced serum CK compared to free SV and preserved normal muscle architecture with minimal degenerative lesions. Furthermore, molecular analyses revealed that FSM-1 treatment normalized the expression of PGC-1α, Mfn1, and Fis1, suggesting restoration of mitochondrial homeostasis and improved cellular bioenergetic balance relative to the free drug group (Fig. 10) [40].

Overall, the study provided strong in vivo evidence that porous microsponges can effectively reduce simvastatin-induced myotoxicity, extending protection beyond morphological and biochemical recovery to include mechanistic correction of mitochondrial-gene dysregulation [40]. Nevertheless, it is notable that the microsponge system operates in the microscale dimension, thereby exhibiting distinct absorption and PK properties from nanocarriers. Additionally, while CK and histological outcomes were indicative of reduced toxicity, the absence of plasma PK parameters such as Cmax, AUC, or direct muscle drug quantification limits mechanistic interpretation regarding whether the reduced toxicity stems from altered systemic exposure or localized gastrointestinal modulation. Given the use of a single species and small sample groups, further studies incorporating chronic dosing, PK–pharmacodynamic correlation, and LDL-lowering efficacy comparisons are required to support translational relevance. Nonetheless, the work convincingly demonstrates that controlled oral release of simvastatin via porous microsponge matrices can attenuate muscle toxicity in vivo, highlighting the importance of mitochondrial biomarkers such as PGC-1α, Mfn1, and Fis1 as sensitive mechanistic endpoints for future statin formulation development.

Co-encapsulating antioxidants (CoQ10, selenium, vitamin E, N-acetylcysteine) directly addresses the mechanistic cause (mitochondrial ROS and CoQ10 depletion). This can be executed in polymeric NPs, lipid NPs, or hybrid carriers [71–77]. Abo-zalam et al. (2024) investigated the use of nanoencapsulated antioxidant systems, solid lipid nanoparticles loaded with either coenzyme Q10 (CoQ10-SLNs) or selenium (Se-SLNs), to mitigate simvastatin-induced myopathy and insulin resistance in a chronic high-fat diet (HFD) rat model [78]. The study utilized a solid lipid nanoparticle platform composed of Compritol, Gelucire, and Poloxamer matrices prepared via hot homogenization followed by ultrasonication, enabling stable, spherical nanocarriers as confirmed by SEM and TEM analysis. Measured by dynamic light scattering, the average particle diameters were 213.9 ± 6.3 nm for CoQ10-SLNs and 296.7 ± 6.3 nm for Se-SLNs, with PDI values of 0.35 and 0.49, respectively, and zeta potentials ranging from − 13.5 to − 6.1 mV, indicating moderate colloidal stability. Entrapment efficiency was remarkably high for both nanocarriers (91.20 ± 2.14% for CoQ10-SLNs and 94.89 ± 1.54% for Se-SLNs), confirming successful loading and entrapment within the lipid matrices [78].

**Fig. 12:**
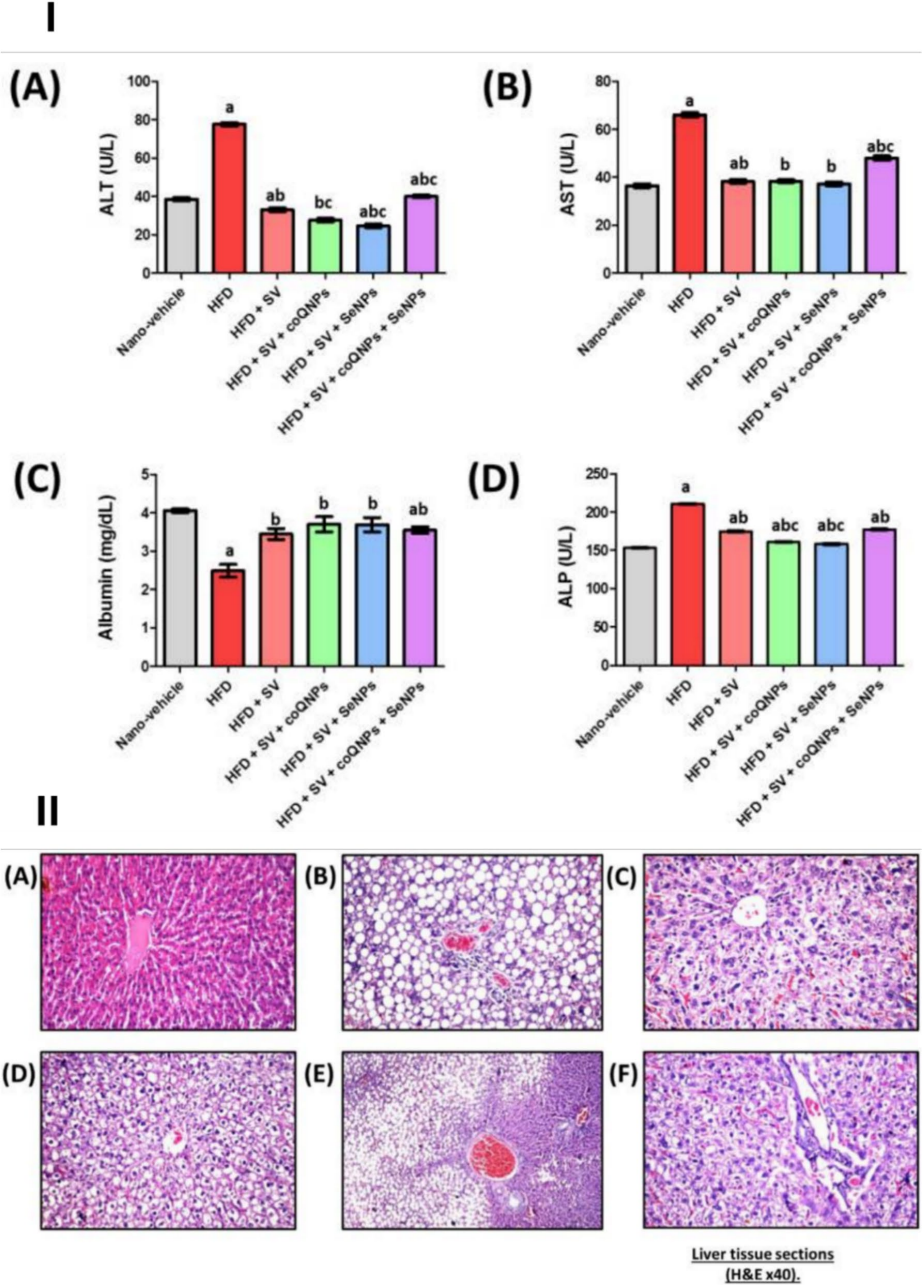
Protective effect of nano‑CoQ10 and nano‑selenium on simvastatin‑induced hepatic alterations in hyperlipidemic rats. (I) Biochemical assessment illustrating (**A**) ALT, (**B**) AST, (**C**) albumin, and (**D**) ALP levels following simvastatin (20 mg/kg) alone or co-loaded with CoQ10 (10 mg/kg) and/or selenium (0.1 mg/kg). (II) Histopathology of liver sections (H&E, × 40): (**A**) nano‑vehicle: slight congestion around central vein and sinusoids; (**B**) HFD: marked portal‑vein congestion, inflammation, and fatty change; (**C**) Simvastatin: diffuse Kupffer‑cell proliferation among degenerated hepatocytes; (**D**) SV + CoQ10 NPs: parenchymal vacuolar degeneration; (**E**) SV + Se NPs: portal‑vein congestion with focal fatty change; (**F**) SV col-loaded with CoQ10 and Se NPs: vacuolar degeneration and mild inflammatory infiltration around portal vein. Reproduced from ref. [78]. Copyright 2024 TUOMS Press

**Fig. 13:**
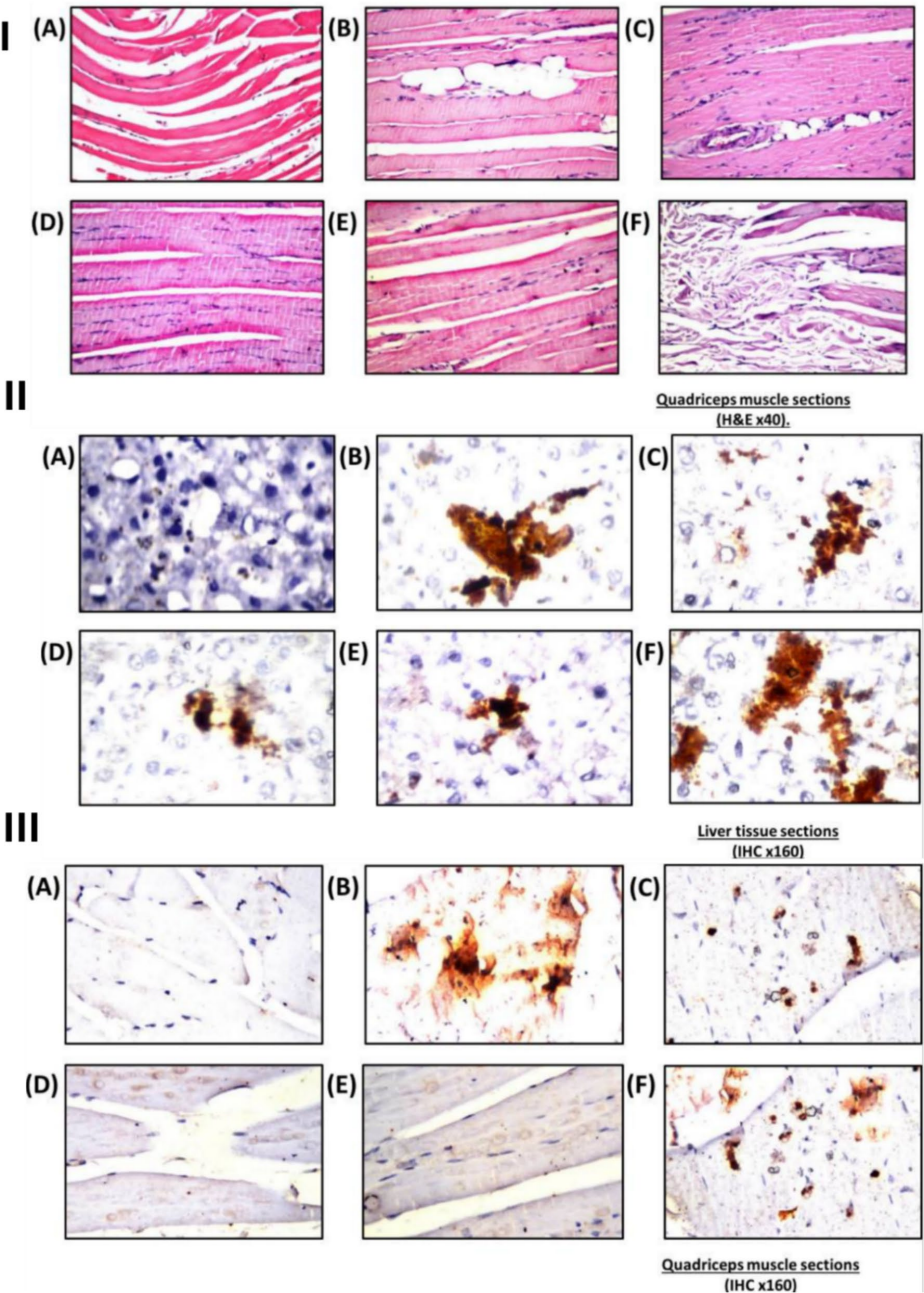
Protective effect of nano‑CoQ10 and nano‑selenium on simvastatin‑induced hepatic and muscular alterations in hyperlipidemic rats. (I) Quadriceps muscle sections (H&E × 40): (**A**) nano-vehicle—typical muscle architecture; (**B**) HFD: fat droplets deposited within atrophied muscle bundles and marked Zenker’s necrosis; (**C**) Simvastatin: focal fat-cell accumulation between bundles with atrophy; (**D**) SV + CoQ10 NPs: normal structure without alterations; (**E**) SV + Se NPs: normal architecture; (**F**) SV + CoQ10 + Se NPs: muscle atrophy accompanied by fat-droplet deposition. (II) Caspase-3 immunostaining in liver sections (H&E × 160): (**A**) nano-vehicle: negative reaction throughout the tissue; (**B**) HFD: strong dark-brown staining indicating increased caspase activity; (**C**) Simvastatin: intense positive reaction; (**D**) SV + CoQ10 NPs: reduced number and intensity of positive cells; (**E**) SV + Se NPs: similar reduction in staining intensity; (**F**) SV + CoQ10 + Se NPs: elevated number and intensity of positive cells suggesting interaction-related toxicity. (III) Caspase-3 immunostaining in quadriceps muscle (H&E × 40): (**A**) nano-vehicle: no positive reaction observed; (**B**) HFD: intense dark-brown staining representing increased caspase activity; (**C**) Simvastatin: similar strong positivity; (**D**) SV + CoQ10 NPs: fewer and weaker positive cells; (**E**) SV + Se NPs: reduced positive cells and staining intensity; (**F**) SV + CoQ10 + Se NPs: higher number and stronger intensity of caspase-positive cells. Reproduced from ref. [78]. Copyright 2024 TUOMS Press

To assess the protective efficacy of these formulations against statin-associated muscle damage, male Wistar rats were subjected to an HFD regimen for 112days, during which groups were assigned to receive HFD alone, HFD plus simvastatin (SV), or SV co-administered with either CoQ10-SLNs, Se-SLNs, or both during the final 30 days of the study. Key endpoints included serum biomarkers of myopathy (CK, myoglobin, troponin-T), muscle histology, oxidative stress parameters (malondialdehyde (MDA), superoxide dismutase (SOD), glutathione (GSH)), liver enzyme activity (ALT/AST), lipid profile, and insulin resistance indices. Quantitative data revealed that serum CK levels were significantly elevated in HFD-alone and HFD + SV groups (226.1 ± 0.95 U/L and 189.9 ± 0.97 U/L, respectively), whereas co-treatment with CoQ10-SLNs or Se-SLNs markedly reduced CK to 168.4 ± 0.98 U/L and 159.3 ± 1.03 U/L, respectively, values approaching the nano-vehicle control group (139.0 ± 0.8 U/L). In contrast, simultaneous administration of SV + CoQ10-SLNs + Se-SLNs resulted in an unexpected elevation of CK (201.4 ± 1.08 U/L), suggesting a potential interaction-induced toxicity when multiple nanoparticle systems are co-delivered. Corroborating this, histopathological analysis demonstrated that SV alone caused notable myofiber disruption and necrosis, whereas co-administration with either CoQ10-SLNs or Se-SLNs restored near-normal muscle architecture with attenuated inflammatory and degenerative changes. However, the triple combination group exhibited worsened tissue injury relative to single-nanoformulation groups, underscoring the need for cautious dosing and compatibility evaluation in multi-agent nanoparticle therapies (Figs. 12 and 13) [79].

Overall, this study provides robust and mechanistically aligned evidence that nanoformulated mitochondrial protectants, when co-administered with simvastatin, can significantly ameliorate biochemical and histological indicators of statin-induced myopathy while maintaining lipid-lowering efficacy. The observed benefits are likely mediated through restoration of redox balance, enhancement of mitochondrial defense pathways, and suppression of oxidative and apoptotic cascades within muscle tissue. Nonetheless, the experimental design involved separate nanoformulations administered concurrently rather than true co-encapsulation, which may partially account for the observed antagonistic effects in the combined group [78]. Moreover, the study did not measure simvastatin PKs or tissue-level drug distribution; thus, the protective interpretation remains mechanistic rather than directly linked to altered bioavailability or reduced intramuscular exposure. Taken together, these findings emphasize that nano-antioxidant co-administration represents a compelling strategy to mitigate statin-associated muscle adverse effects, yet future studies should pursue co-encapsulated SV + antioxidant designs paired with PK and tissue-distribution profiling to establish a causal link between formulation, exposure, and toxicity reduction.

**Fig. 14:**
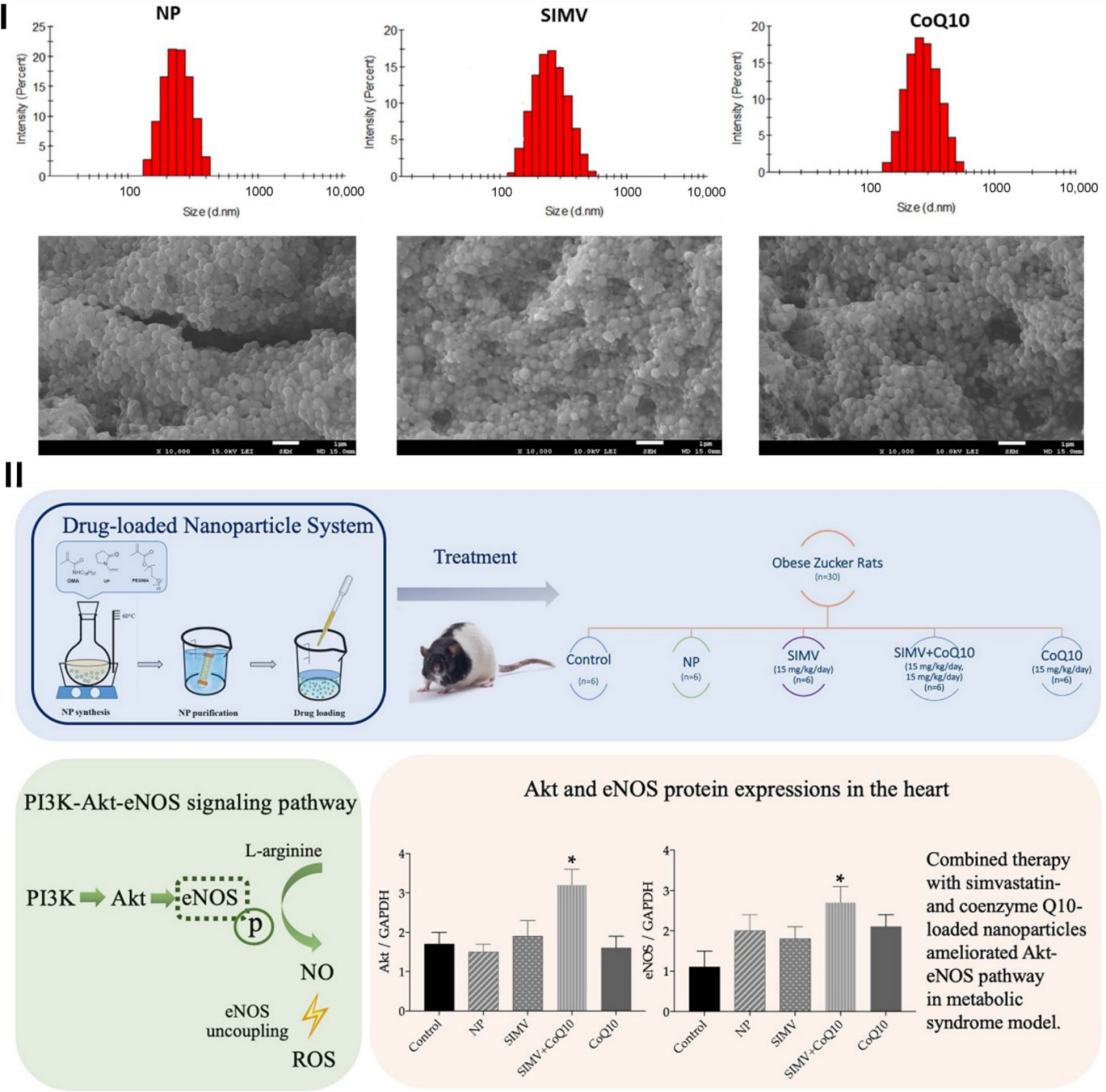
(I) DLS characterization and SEM spectroscopy of unloaded NPs and their corresponding NPs-loaded with simvastatin or CoQ10. (II) Graphical summary of the combined treatment with simvastatin- and coenzyme Q10-loaded polymeric nanoparticles (SIMV + CoQ10 NPs) in obese Zucker rats. The NPs enhanced cardiovascular function by upregulating the Akt–eNOS pathway in the heart and aorta, which increased nitric oxide (NOS) activity and endothelial signaling, thereby improving vascular relaxation. This combination also reduced oxidative stress markers such as NADPH oxidase and NF-κB, leading to attenuated inflammation and oxidative damage. Free simvastatin or CoQ10 NPs alone offered partial benefit, whereas the co-delivered form demonstrated synergistic activation of eNOS and energy-balance pathways. Overall, the graphical abstract illustrates how SIMV + CoQ10 NPs restore the NO/ROS equilibrium and support cardioprotective mechanisms in metabolic syndrome. Reproduced from ref. [47]. Copyright 2022 MDPI

Moreover, Şaman et al. (2023) developed dual nanoparticle systems based on amphiphilic polymeric carriers to deliver simvastatin (SIMV-NP) and coenzyme Q10 (CoQ10-NP) either individually or in combination, aiming to modulate endothelial and oxidative stress pathways implicated in metabolic syndrome–associated vascular dysfunction [47]. The nanoparticles were fabricated via a nanoprecipitation/diffusion technique, yielding spherical polymeric nanocarriers as confirmed by DLS and SEM analysis. The mean hydrodynamic diameters were approximately 233 ± 14 nm for the unloaded nanoparticles, 347 ± 52 nm for SIMV-NPs, and 270 ± 24 nm for CoQ10-NPs, with reported polydispersity indices (0.03, 0.23, and 0.12, respectively) and zeta potential values (− 15.5, − 29.2, and − 15.8, respectively) indicating moderate colloidal uniformity and stability (Fig. 14). Drug loading and encapsulation efficiencies for both simvastatin and CoQ10 were optimized to ensure physiologically relevant concentrations and sustained release kinetics throughout the treatment duration [47].

**Fig. 15:**
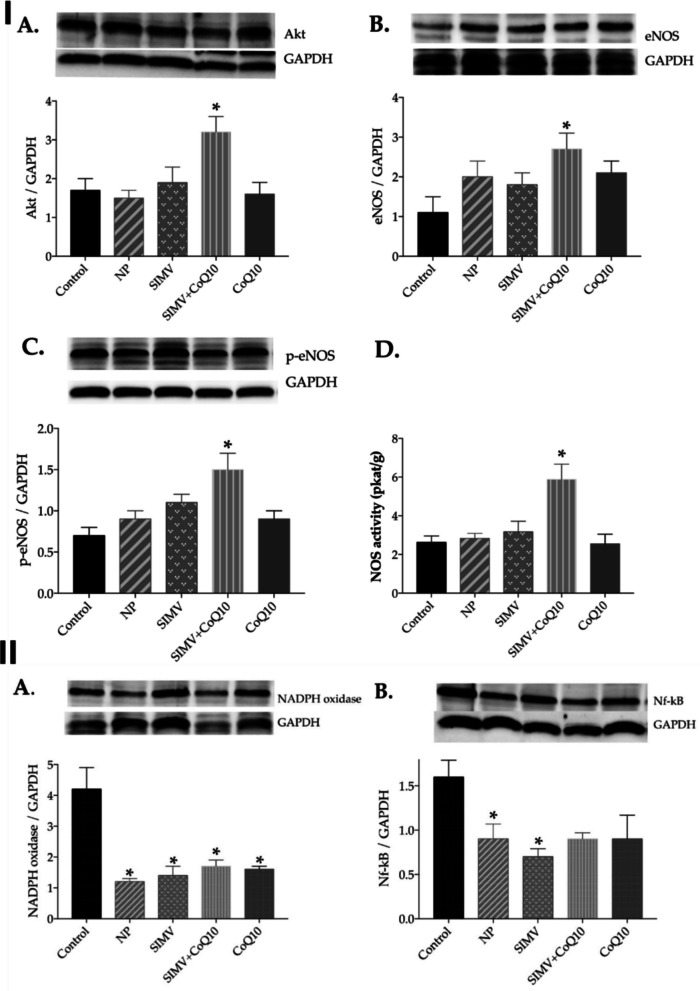
Effect of simvastatin- and coenzyme-Q10-loaded nanoparticles on endothelial and oxidative signaling in obese Zucker rats. (I) Expression levels and representative Western blot images of (**A**) Akt, (**B**) endothelial nitric oxide synthase (eNOS), (**C**) phosphorylated eNOS (p-eNOS), and (**D**) total nitric oxide synthase (NOS) activity in cardiac tissue of control rats and animals treated with empty NPs, simvastatin-loaded nanoparticles (SIMV), combined simvastatin- and coenzyme-Q10-loaded nanoparticles (SIMV + CoQ10), or coenzyme-Q10-loaded nanoparticles (CoQ10). (II) Expression levels and representative Western blots of (**A**) nicotinamide adenine dinucleotide phosphate (NADPH) oxidase and (**B**) nuclear factor-κB (NF-κB) in the heart of control and treated rats. Reproduced from ref. [47]. Copyright 2022 MDPI

**Fig. 16:**
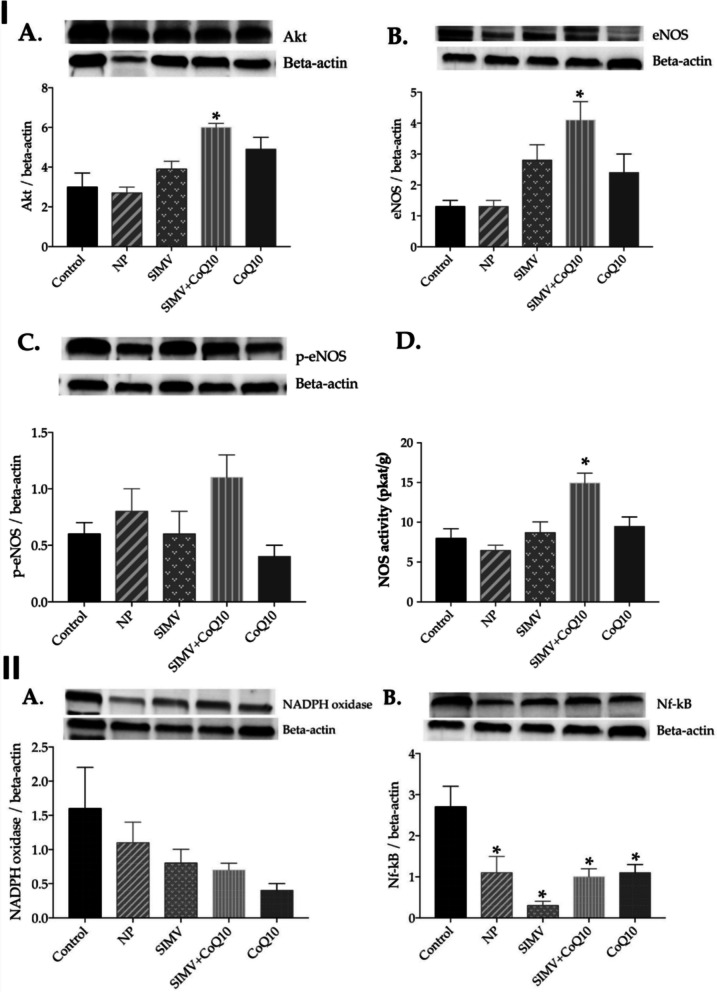
Effect of simvastatin- and coenzyme-Q10-loaded nanoparticles on endothelial and oxidative signaling in obese Zucker rats. (I) Expression levels and representative Western blot images of (**A**) Akt, (**B**) eNOS, (**C**) p-eNOS, and (**D**) total NOS activity in aortic tissue of control and treated rats. (II) Expression levels and representative Western blots of (**A**) NADPH oxidase and (**B**) NF-κB in aortic tissue of control and treated rats. Reproduced from ref. [47]. Copyright 2022 MDPI

The study employed an obese Zucker rat model representative of metabolic syndrome, characterized by hyperlipidemia, insulin resistance, and endothelial oxidative imbalance. Animals were allocated to multiple treatment study groups receiving either single SIMV-NP, single CoQ10-NP, or combined SIMV-NP + CoQ10-NP therapy, alongside appropriate free-drug and untreated controls, over a defined 6-week dosing schedule. Endpoints focused on vascular oxidative and inflammatory pathways, with particular emphasis on the Akt–eNOS signaling cascade, nitric oxide (NO) regulation, and reactive oxygen species (ROS) production. Biochemical and molecular analyses revealed that while either SIMV-NP or CoQ10-NP improved oxidative status and lipid parameters individually, the combined nanoparticle therapy produced the most pronounced effects. Specifically, co-delivery significantly enhanced endothelial NO synthase (eNOS) phosphorylation through Akt pathway activation, increased total NOS activity, and suppressed NADPH oxidase and NF-κB expression, collectively indicative of reduced vascular oxidative stress and improved endothelial bioactivity (Fig. 15 and 16). The results support a mechanistic synergy between statin therapy and antioxidant supplementation when co-delivered via nanoparticle systems, restoring endothelial signaling balance disrupted under metabolic syndrome conditions [47].

Although the study did not include classical skeletal muscle myotoxicity endpoints such as serum CK levels or muscle histopathology, its findings provide valuable mechanistic context relevant to statin-associated muscle effects. By demonstrating enhanced mitochondrial and NO-mediated signaling and attenuation of ROS stress under combined nanoparticle delivery, the work implicates a mechanistic route through which co-administration of CoQ10 may also confer protective benefit against statin-related oxidative injury [47]. However, the absence of PK, biodistribution, and myopathy-specific data limits direct conclusions regarding SAM mitigation. Overall, this study complements other nanoparticle-based co-delivery research by corroborating that concurrent nanoformulation of simvastatin with an antioxidant cofactor can beneficially remodel oxidative and endothelial signaling networks, highlighting the translational potential of such designs for integrated cardiometabolic and mitochondrial protection, pending future inclusion of skeletal muscle–specific endpoints.

The following table (Table 2) summarizes the Key findings of the nanosystems utilized for loading simvastatin to mitigate its induced myopathy along to the main characteristics of the obtained nanoformulations.

**Table 2:** Key primary studies of Simvastatin-loaded nanosystems relevant to mitigation of myopathy

Platform/nanocarrier	Particle size/properties	Model (in vitro/in vivo)	Dose/regimen	Myopathy/muscle endpoints measured	Key findings (muscle/CK/histology etc.)	Remarks
Hyaluronic acid-based polymeric NPs [39]	~ 280 nm, − 25.6 mV	3D collagen-based tissue-engineered skeletal muscle (in vitro)	Concentrations: 33.33 nM, 3.33 µM, 333.33 µM	Contractile function, viability, cytoskeletal integrity, apoptosis markers	HA-SIM NPs preserved contractile function and morphology better than free drug	Strong mechanistic tissue model, but lacks in vivo translation
Eudragit RS-100 microsponges [40]	53.80–86.03 µm microscale	In vivo (Wistar rats)	Oral dosing (20 mg/kg/day), SIM vs. SIM-microsponges groups	Serum CK, muscle histology, mitochondrial gene expression (PGC-1α, Mfn1, and Fis1)	Microsponges reduced CK, improved histology, better mitochondrial gene profiles vs free drug	Animal evidence of reduced myotoxicity, but no PK investigation
SLNs [41]	255 ± 7.7 nm, PDI 0.31 ± 0.09, zeta potential − 19.30 ± 3.25 mV, and EE% 89.81 ± 2.1%	In vivo (Wistar albino rats with high-fat diet)	Free simvastatin 20 mg/kg, SLNs 20 mg/kg + low-dose SLNs 5 mg/kg	CK, muscle histology, apoptosis (caspase-3), oxidative stress	SLNs groups showed lower muscle damage histologically, lower CK, improved markers; low dose group normalized many endpoints	Strong in vivo evidence with metabolic disease model; needs PK/tissue distribution proof
Nano-CoQ10/Selenium NPs + SIM combinations. [78]	CoQ10-SLNs: 213.9 ± 6.3 nm, PDI 0.35, EE% 91.20 ± 2.14% Se-SLNs: 296.7 ± 6.3 nm, PDI 0.49, EE% 94.89 ± 1.54% Zeta potentials ranging from − 13.5 to − 6.1 mV	In vivo (male Wistar rats)	Co-treatment with SIM + CoQ10 NPs or SeNPs	CK, histology, mitochondrial function	Co-delivery groups reduced CK, better muscle/mitochondrial markers than SIM alone	In vivo evidence of co-encapsulation with mitochondrial protectants efficiency; still lacks PK studies and further investigation on drug tissues distribution
Polymeric NPs co-loaded with simvastatin + CoQ10 [47]	SIM NPs: 347 ± 52 nm, PDI 0.23, zeta potential − 29.2 CoQ10 NPs: 270 ± 24 nm, PDI 0.12, zeta potential − 15.8	In vivo (endothelial and cardiac protection in obese Zucker rats)	SIM NPs, CoQ10 NPs, SIM + CoQ10 NPs (6 weeks)	No direct muscle toxicity endpoints; measured lipid profile, ROS/NO balance, Akt/eNOS signaling in heart/aorta	No CK, muscle histology, or muscle drug levels reported	No signs of muscle damage or CK elevation were reported

*NPs* nanoparticles, *HA* hyaluronic acid, *SIM* Simvastatin, *CK* creatine kinase, *PGC-1α* peroxisome proliferator-activated receptor gamma co-activator 1α, *Mfn1* mitochondrial fusion protein, *Fis1* mitochondrial fission protein, *PK* pharmacokinetic, *SLNs* solid lipid nanoparticles, *CoQ10* coenzyme Q10, *NO* nitric oxide, *eNOS* endothelial nitric oxide synthase, *ROS* reactive oxygen species, *SeNPs* selenium nanoparticles.

Another promising candidate among the statin drugs that has further been briefly investigated is atorvastatin. Atorvastatin, like simvastatin, is lipophilic and subject to first-pass metabolism [79–83]. Therefore, several nanoparticulate approaches have been widely explored to improve oral bioavailability, prolong exposure and, in some studies, reduce off-target toxicity of atorvastatin. Meanwhile, few representative preclinical reports suggest that atorvastatin formulated as nanocrystals or SLNs can preserve antihyperlipidemic efficacy at lower doses and are associated with reduced biochemical and histological indices of hepatic and muscular injury when compared with free drug, especially when combined with mitochondrial antioxidants (e.g., CoQ10, vitamin E) [84, 85].

**Fig. 17:**
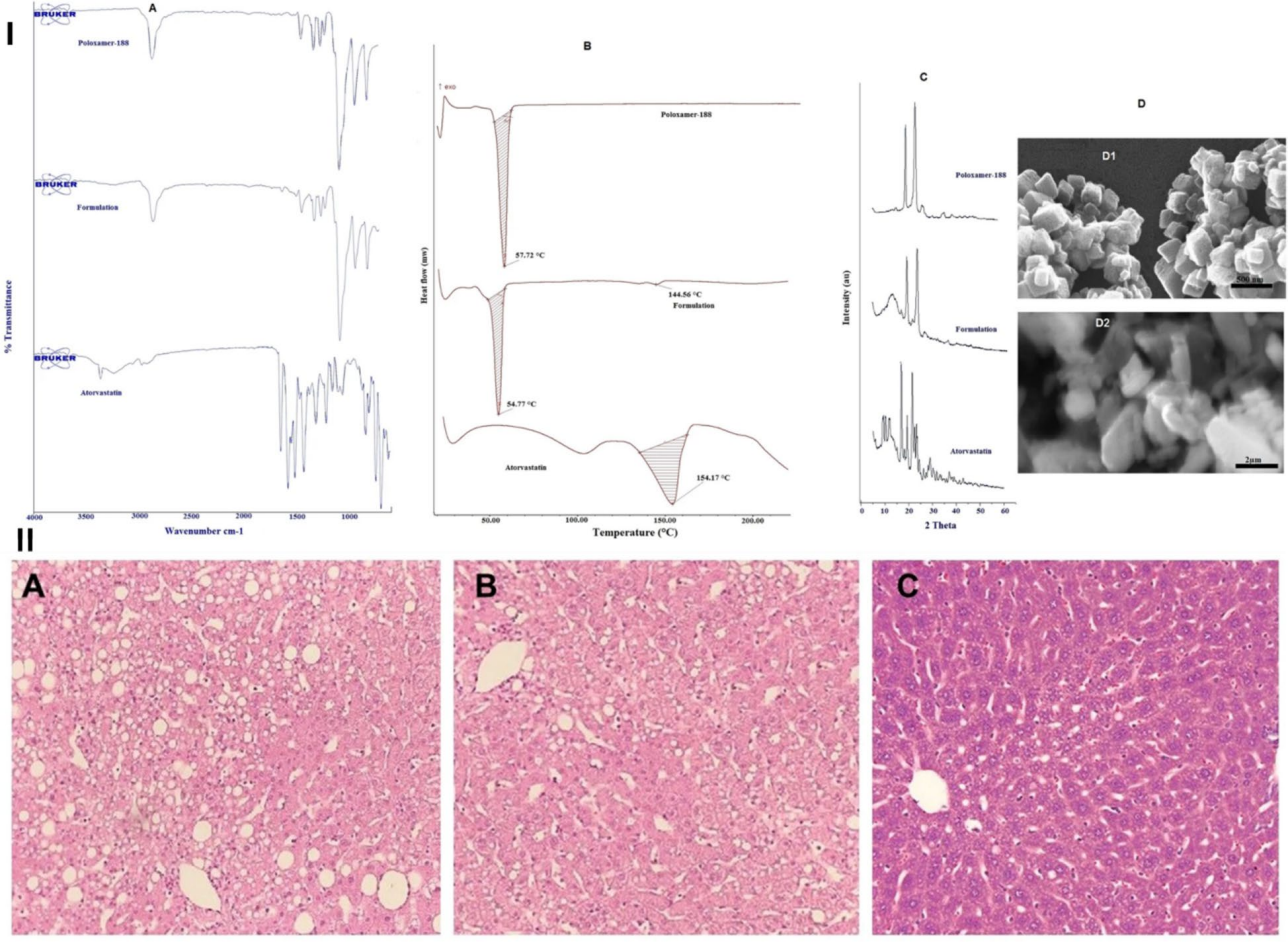
(I) Analytical profiles and morphology: ATR-FTIR analysis (**A**) comparing atorvastatin, poloxamer 188, and atorvastatin-loaded nanocrystals (formulation); DSC studies (**B**) confirming stable drug-excipient interactions and modified melting behavior in nanocrystals; Powder XRD (**C**) revealing reduced crystallinity in nanocrystals due to poloxamer surface coverage, supporting enhanced solubility; SEM micrographs (**D**) showing cubical nanoscale particles for the optimized formulation (D1) versus larger crystals in the pure drug (D2). (II) Liver histopathology: hepatic morphology in (**A**) high-fat diet control, (**B**) free drug-treated, and (**C**) nanocrystal-treated groups, demonstrating reduced fat accumulation and improved architecture in rats receiving the optimized formulation. Reproduced from ref [84]. Copyright 2019 Nature

For instance, Sharma and Mehta (2019) developed surface-stabilized atorvastatin nanocrystals using poloxamer-188 and high-pressure homogenization, resulting in a uniform cubical morphology with particle sizes of 170–240 nm (as shown by SEM) and a narrow size distribution (Fig. 17) [84]. These nanocrystals demonstrated dramatically enhanced aqueous solubility, roughly 18-fold in water and up to 40-fold at gastric pH compared to the raw drug. Following oral administration in Wistar rats, PK profiling showed that the nanocrystal formulation led to a 2.66-fold increase in total atorvastatin bioavailability (AUC_0–24_) and a 2.19-fold higher peak plasma concentration compared to the conventional suspension, along with a lower T_max_ and prolonged elimination half-life and mean residence time (MRT increased by 1.42-fold). Most notably, the nanocrystal group achieved equivalent lipid-lowering effects at just half the dose of the conventional drug, with total cholesterol, LDL, VLDL, and triglycerides all reduced to near normal levels after 2 weeks of treatment. In parallel, key safety markers related to the muscles in the treated rats with both free drug and nanocrystals were measured. While plasma levels of CK showed insignificant change, other biomarkers such as urea, creatinine, and lactate dehydrogenase exhibited significantly lower levels. Meanwhile, histopathological analysis revealed less hepatic fat accumulation and vascular abnormality following treatments with nanocrystals (Fig. 17). These findings highlight that nanocrystal-based delivery not only boosts atorvastatin absorption and efficacy at lower doses, but also confers a superior safety margin, likely due to improved dissolution, slower elimination, and reduced off-target accumulation supporting the clinical promise of statin nanoformulations for hyperlipidemia management [84].

**Fig. 18:**
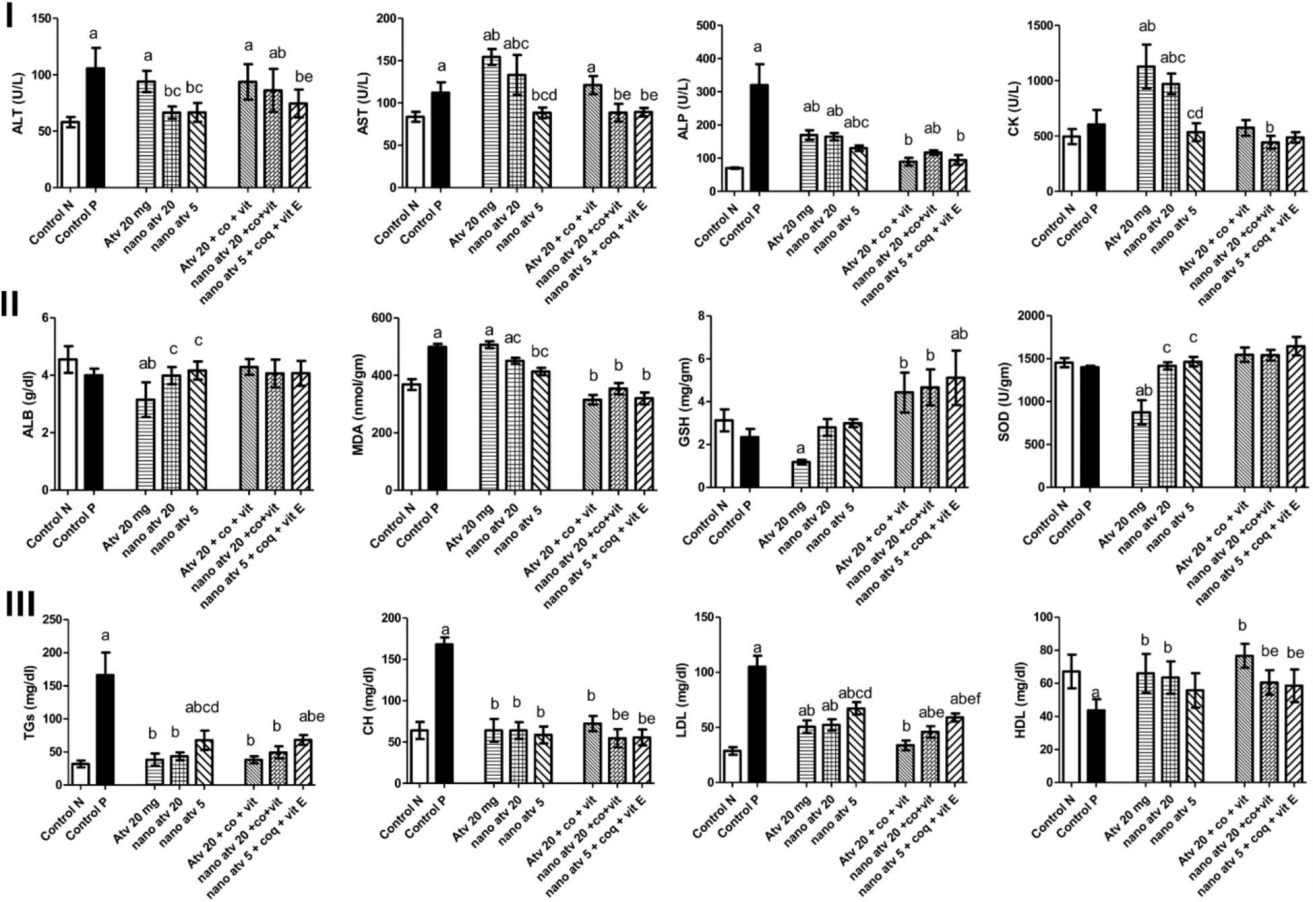
Effects of different atorvastatin formulations, alone and in combination with coenzyme Q10 and vitamin E, on liver and muscle enzyme levels, albumin, oxidative stress biomarkers, and lipid profile in hyperlipidemic rats. Superscripts indicate significant differences: (**a**) versus normal control, (**b**) versus hyperlipidemic control, (**c**) versus free atorvastatin (20 mg/kg), (**d**) versus nanoparticle atorvastatin (20 mg/kg), (**e**) versus free drug combination, (f) versus nanoparticle drug combination. Reproduced and modified with permission from ref [85]. Copyright 2018 Elsevier

**Fig. 19:**
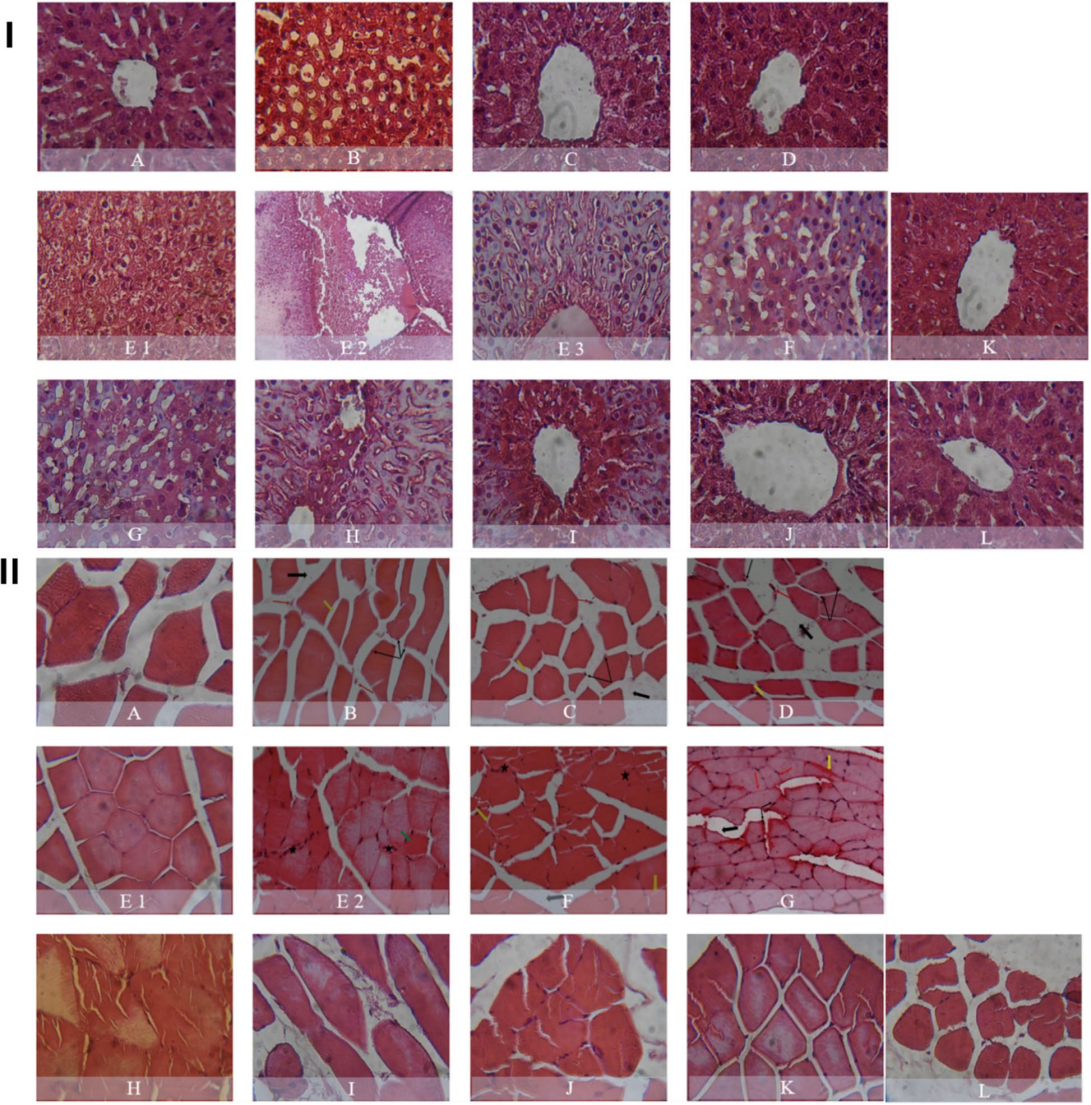
Representative histopathological images of liver (I) and quadriceps muscle (II) from rats treated with different atorvastatin, coenzyme Q10, and vitamin E formulations in both free and nanoparticle forms. Panels correspond to (**A**) normal control, (**B**) hyperlipidemic control, (**C**) coenzyme Q10, (**D**) vitamin E, (E1, E2, E3) free atorvastatin (20 mg/kg), (**F**) free atorvastatin + coenzyme Q10, (**G**) free atorvastatin + vitamin E, (**H**) free atorvastatin + coenzyme Q10 + vitamin E, (**I**) nanoparticle atorvastatin (20 mg/kg), (J) nanoparticle atorvastatin + coenzyme Q10 + vitamin E, (**K**) nanoparticle atorvastatin (5 mg/kg), (**L**) nanoparticle atorvastatin (5 mg/kg) + coenzyme Q10 + vitamin E. Reproduced and modified with permission from ref [85]. Copyright 2018 Elsevier

Furthermore, Farrag et al. (2018) directly addressed the toxicity in a hyperlipidemic rat model (Wistar rats) by comparing atorvastatin in SLNs formulation alone or co-administered with antioxidant supplements (coenzyme Q10 and/or vitamin E) [85]. After 12 weeks of a high-fat diet, rats received oral atorvastatin in either conventional or nanoparticle form, at 5 or 20 mg/kg/day, with or without coenzyme Q10 (10 mg/kg/day) or vitamin E (30 mg/kg/day), for four further weeks. Compared to the free drug, nanoparticle-based atorvastatin led to significantly reduced markers of liver and muscle injury: ALT decreased to 93.9 and 82.67 U/L at the higher and lower nanoparticle doses, versus 105.6 U/L with the hyperlipidemic diet; AST was lowered to 121.1 U/L in the nanoformulation with antioxidants, compared to 154.2 U/L in the free drug group; and CK dropped from 1129 U/L (free atorvastatin) to 615.6 and 574.2 U/L in the nanoparticle-treated groups. In addition, rats receiving the combined nanoformulation with coenzyme Q10 and vitamin E exhibited elevated GSH concentration to 4.42 μmol/g tissue, more than twice the levels in the untreated hyperlipidemic group, and 1.5 times those in healthy controls. Markers of oxidative stress improved as well: MDA fell to 315.3 nmol/g tissue in the nanoparticle-treated group with antioxidants, compared to 499 nmol/g in hyperlipidemic controls, while SOD increased to 1387–1546 U/g tissue in nanoparticle and antioxidant-treated rats, far above the values in free drug-treated animals (Fig. 18). Muscle and liver tissue analysis further confirmed the biochemical changes: nanoparticle-based atorvastatin, especially with added antioxidants, preserved normal architecture and minimized vacuolation and inflammation, whereas standard statin therapy led to marked tissue degeneration. These findings demonstrate that nano-statins, especially in combination with coenzyme Q10 and vitamin E, can significantly reduce liver and muscle toxicity compared to conventional statin therapy (Fig. 19). These findings can be attributed to the improved formulation kinetics (sustained release/altered absorption) together with the mitochondrial antioxidant support which reduced drug-induced oxidative injury [85].

Other approaches employing SLNs, NLCs, nanospheres, and polymeric nanoparticles for atorvastatin delivery have consistently shown benefits including enhanced dissolution, greater oral bioavailability, potential for dose reduction, and reduced organ toxicity in preclinical models. Importantly, these studies highlight the need for systematic PK and toxicological evaluation pipelines to establish direct causality, specifically, to demonstrate that reductions in plasma and muscle exposure to statins translate directly to improvements in biochemical markers (such as CK) and histopathological outcomes. Rigorous, standardized methods are critical for substantiating the mechanistic link between targeted drug delivery and mitigation of statin-induced myopathy [85–95].

Pitavastatin has uniquely advanced to clinical nanoparticle programs for local intramuscular delivery aimed at therapeutic angiogenesis in critical limb ischemia [96]. Matsumoto et al. (2022) conducted a phase I/IIa open-label clinical trial to evaluate the safety and therapeutic potential of pitavastatin-incorporated PLGA nanoparticles (NK-104-NP) in patients with chronic limb-threatening ischemia (CLTI). Sixteen patients received intramuscular injections of NK-104-NP at escalating doses (0.5, 1, 2, and 4 mg pitavastatin calcium; *n* = 4 each) for five consecutive days into the ischemic limb, followed by 26-week monitoring for safety and efficacy. The treatment was well tolerated with no statin-related toxicity or serious adverse events. Improvement in limb ischemia status (assessed by Fontaine and Rutherford classifications) was documented in five participants across the short-term follow-up period. PK analysis showed a dose-dependent increase in systemic pitavastatin exposure (Cmax and AUC) without evidence of drug accumulation over the 5-day administration. The study concluded that intramuscular delivery of pitavastatin via PLGA nanocarriers is safe and potentially therapeutic for enhancing limb perfusion and function in CLTI patients [96]. Hence, this work showed NK-104-NP intramuscular injections were safe and improved limb outcomes without serious systemic adverse events in clinical phase I/IIa trials. However, these programs focus on local, not systemic, delivery and therefore do not directly address systemic statin myopathy reduction, but they are an important translational precedent for statin-NP safety in humans.

On the other hand, numerous nanoformulations have been developed for various statin agents beyond simvastatin and atorvastatin, including pravastatin, rosuvastatin, lovastatin, fluvastatin, and others [44, 51, 97–106]. These have employed a range of carriers with documented aims including improved oral bioavailability, enhanced PKs, targeted tissue or vascular delivery, and, in some cases, co-delivery of other bioactives or site-specific therapeutic modulation. However, a critical review of the literature reveals that, despite robust evidence for improved dissolution and pharmacological efficacy, there is a remarkable paucity of studies that have evaluated these nanoformulations with the explicit goal of reducing systemic statin myopathy or rigorously measured muscle toxicity endpoints, such as serum CK or muscle histology, for agents other than simvastatin and atorvastatin. This gap highlights a clear need for future studies: the successful nanoformulation strategies developed for simvastatin and atorvastatin, including tissue targeting, PK optimization, and use of antioxidant adjuncts, should be systematically extended to other statins. Importantly, standardized protocols for PK and muscle toxicity assessment are essential to directly link exposure modulation with reduction of statin-induced muscle adverse effects.

## Critical appraisal and translational perspectives

This review primarily highlighted the preclinical evidence that nanoformulations of simvastatin can mitigate statin-associated myopathy. Across multiple platforms, hyaluronan-based polymeric nanoparticles, porous microsponges, SLNs, and co-delivery nanosystems containing mitochondrial protectants, investigators observed reductions in key myotoxicity markers, including serum CK, histological muscle damage, apoptosis markers, and impaired contractile function [39–41, 78]. These results support two mechanistic concepts previously described. First, modulation of PK (lower Cmax via sustained release or hepatic bias) can reduce peak muscle exposure to lipophilic statins. Second, biochemical protection (antioxidant or CoQ10 supplementation) can minimize mitochondrial dysfunction and ROS-mediated injury.

Despite promising proof-of-concept results, there remains a critical translation gap. None of the current simvastatin studies provide a full PK and biodistribution chain-of-evidence demonstrating that the nanoformulation reduced plasma Cmax or muscle tissue concentrations of simvastatin in vivo. Most studies infer the mechanism by combining controlled-release formulations with improved muscle endpoints; however, without quantitative evidence of reduced muscle drug levels (for example, LC–MS/MS measurements of simvastatin in skeletal muscle or serial PK sampling showing reduced Cmax with similar or preserved hepatic exposure), causality remains unproven. Filling that gap should be a primary objective of future preclinical studies.

A second translational challenge is co-therapy complexity. The Abo-zalam (2024) [78] study demonstrated that nano-CoQ10 or Se-SLNs given with simvastatin each reduce myotoxicity markers, but a triple combination unexpectedly worsened outcomes at the tested dosing ratios. This highlights that multi-agent nano-regimens require careful dose-finding and safety testing to avoid unforeseen interactions.

Compared to simvastatin, other statins have been less thoroughly evaluated for NPs-mediated myopathy mitigation. Atorvastatin, pitavastatin, pravastatin, rosuvastatin, lovastatin, and fluvastatin have many nanoparticulate formulations reported in the literature, but very few include dedicated myotoxicity endpoints. Pitavastatin stands out for having advanced translational work (e.g., intramuscular NK-104 clinical NP programs for limb ischemia) [96], but none specifically tested reduction of systemic statin myopathy. This gap represents an opportunity, in which the conceptual strategies validated with simvastatin, controlled release, hepatic targeting, and co-delivery, are broadly applicable and should be tested across other statins, especially those with lipophilic properties that predispose to muscle exposure.

While hepatic targeting is a central objective of statin nanocarrier design, potential implications for liver safety require careful consideration. Although statin-induced hepatotoxicity is less prevalent than myopathy, clinically relevant elevations in liver enzymes and rare cases of drug-induced liver injury have been documented [107–112]. Several nanoformulation studies reviewed herein reported normalization or improvement of hepatic biochemical markers relative to free statins, likely reflecting dose-sparing effects and reduced systemic exposure.

Nevertheless, enhanced hepatic accumulation may pose long-term risks not captured in short-duration preclinical investigations. Comprehensive assessment of chronic hepatocellular stress, bile acid homeostasis, and histopathological changes is essential as nanocarrier strategies progress toward clinical translation. Balanced safety evaluation across skeletal muscle and hepatic tissues should remain a defining criterion of next-generation statin nanomedicine.

Finally, it is of uttermost important to develop a standardized preclinical approach for evaluating statin-loaded nanosystems intended to reduce myopathy: (1) full physicochemical characterization (size, PDI, zeta potential, EE, release kinetics); (2) in vitro screening in human-relevant skeletal muscle models (2D + 3D, organoids, tissue-engineered muscle) with functional endpoints; (3) in vivo PK and biodistribution studies including quantification of drug in plasma, liver and skeletal muscle (LC–MS/MS), along with CK and histology; (4) demonstration that LDL-lowering efficacy is preserved; and (5) chronic safety studies and dose-finding for co-delivery regimens. Standardization along these lines will make future comparisons meaningful and accelerate clinical translation.

An alternative pharmacological strategy that reinforces the rationale for hepatic-selective statin delivery is bempedoic acid (BPA), a first-in-class ATP-citrate lyase inhibitor developed for lipid lowering in statin-intolerant patients. BPA is a prodrug activated exclusively in the liver due to the absence of the activating enzyme very-long-chain acyl-CoA synthetase-1 in skeletal muscle, thereby minimizing muscular exposure and reducing the risk of myopathy. Clinical trials have demonstrated effective LDL-C reduction with improved tolerability among individuals unable to tolerate conventional statin therapy [113–117].

Theoretically, the muscle-sparing pharmacology of BPA parallels nanocarrier-based statin strategies, as both aim to confine lipid-lowering activity to hepatic tissues while limiting systemic and muscular drug exposure. However, while BPA achieves selectivity via metabolic activation, nanocarrier systems offer additional flexibility by enabling controlled release, biodistribution modulation, and co-delivery of mitochondrial protectants. Long-term data directly evaluating muscle outcomes with BPA remain limited, highlighting the continued relevance of delivery-based approaches for optimizing statin safety.

Overall, nanoformulation offers a promising strategy to decrease statin myotoxicity and widen safe use of an otherwise life-saving drug class. Simvastatin is the best-studied example to date, while expanding rigorous PK-linked preclinical testing to other statins is the next logical step toward clinical translation.

## Conclusions and future perspectives

Nanocarrier-based reformulation of statins represents a promising strategy to mitigate statin-associated myopathy by modulating pharmacokinetics, limiting skeletal muscle exposure, and counteracting mitochondrial dysfunction and oxidative stress. Among these, simvastatin nanoformulations, such as hyaluronan-based carriers, solid lipid nanoparticles, and porous microsponges, consistently demonstrate reduced myotoxicity markers, preserved muscle architecture, and improved functional outcomes compared with free drug administration. Importantly, emerging co-delivery approaches incorporating mitochondrial protectants, including coenzyme Q10 or selenium, further strengthen mechanistic protection against statin-induced muscle injury. However, current evidence remains largely preclinical, with limited integration of standardized pharmacokinetic tissue distribution analyses and minimal assessment of long-term hepatic safety or regenerative endpoints.

Future investigations should prioritize quantitative biodistribution profiling, incorporation of muscle regeneration markers, and comprehensive multi-organ safety evaluation to enable rational clinical translation. Collectively, nanocarrier strategies offer a versatile platform to reengineer statin therapy toward enhanced efficacy and tolerability, potentially transforming lipid-lowering treatment paradigms for statin-intolerant populations.

## Data Availability

No datasets were generated or analysed during the current study.
